# Antigenic cartography of immune responses to *Plasmodium falciparum* erythrocyte membrane protein 1 (PfEMP1)

**DOI:** 10.1371/journal.ppat.1007870

**Published:** 2019-07-01

**Authors:** James Tuju, Margaret J. Mackinnon, Abdirahman I. Abdi, Henry Karanja, Jennifer N. Musyoki, George M. Warimwe, Evelyn N. Gitau, Kevin Marsh, Peter C. Bull, Britta C. Urban

**Affiliations:** 1 KEMRI-Wellcome Trust Research Programme, Kenya; 2 Department of Chemistry and Biochemistry, Pwani University, Kilifi, Kenya; 3 Centre for Tropical Medicine and Global Health, Nuffield Department of Medicine, University of Oxford, Oxford, United Kingdom; 4 African Population and Health Research Center, Nairobi, Kenya; 5 Liverpool School of Tropical Medicine, Department of Tropical Disease Biology, Pembroke Place, Liverpool, United Kingdom; University of Copenhagen and Rigshospitalet, Copenhagen, DENMARK

## Abstract

Naturally acquired clinical immunity to *Plasmodium falciparum* is partly mediated by antibodies directed at parasite-derived antigens expressed on the surface of red blood cells which mediate disease and are extremely diverse. Unlike children, adults recognize a broad range of variant surface antigens (VSAs) and are protected from severe disease. Though crucial to the design and feasibility of an effective malaria vaccine, it is not yet known whether immunity arises through cumulative exposure to each of many antigenic types, cross-reactivity between antigenic types, or some other mechanism. In this study, we measured plasma antibody responses of 36 children with symptomatic malaria to a diverse panel of 36 recombinant proteins comprising part of the DBLα domain (the ‘DBLα-tag’) of PfEMP1, a major class of VSAs. We found that although plasma antibody responses were highly specific to individual antigens, serological profiles of responses across antigens fell into one of just two distinct types. One type was found almost exclusively in children that succumbed to severe disease (19 out of 20) while the other occurred in all children with mild disease (16 out of 16). Moreover, children with severe malaria had serological profiles that were narrower in antigen specificity and shorter-lived than those in children with mild malaria. Borrowing a novel technique used in influenza–antigenic cartography—we mapped these dichotomous serological profiles to amino acid sequence variation within a small sub-region of the PfEMP1 DBLα domain. By applying our methodology on a larger scale, it should be possible to identify epitopes responsible for eliciting the protective version of serological profiles to PfEMP1 thereby accelerating development of a broadly effective anti-disease malaria vaccine.

## Author summary

Immunity to human malaria parasites never fully protects against infection, even after a lifetime of exposure. By contrast, protection against severe disease occurs early in life in malaria-endemic areas. Both anti-infection and anti-disease immunity depend on antibody responses to proteins expressed by the parasite on the red blood cell surface which cause pathology. These proteins are extremely diverse thus creating a problem for designing a widely effective vaccine. Despite its importance to vaccine design, however, it is not yet known whether protection against malaria depends on accumulation of exposure to each of the many antigenic types, or whether this is accelerated due to cross-reactivity between antigenic types or some other mechanism. In this study, by applying a novel technique used for describing antigenic diversity in influenza viruses–antigenic cartography—we make the surprising discoveries that children’s serological responses to a panel of diverse surface antigens fall into one of just two qualitatively distinct patterns, and that these almost perfectly predict severity of disease. These alternative serological profiles were found to associate with genetic variants within a small region of the protein. By using the methodology presented here to define the antigenic types and their underlying genetic variants that give rise to the protective version of the serological response, progress towards development of a broadly protective vaccine against severe malaria might be accelerated.

## Introduction

The surface of red blood cells (RBCs) infected with *Plasmodium falciparum* contains antigens of parasite origin that are highly immunogenic and genetically very diverse [1]. Diversity in variant surface antigens (VSAs) plays an important role in immune evasion and thus in prolonging infections: this affords parasites more opportunities to transmit to new hosts. Acquisition of antibodies to the most studied family of VSAs–*P*. *falciparum* erythrocyte membrane protein 1 *(*PfEMP1)—is associated with protection against malarial disease [2,3]. Since PfEMP1 also plays a key role in pathology due to its property as an adhesion ligand to host cells [4], this protein family makes an attractive target for vaccine development.

PfEMP1 is encoded by approximately 60 *var* genes which are genetically diverse within and between parasite genomes, and which recombine, thus potentially presenting a challenge in finding a handful of antigens that could form the basis of a broadly effective vaccine. Despite their genetic diversity, however, *var* genes structure into distinct groups thus somewhat limiting this pool of variability. These groupings are based on chromosomal position and upstream sequence (Ups) [5,6]; combinations of domains and sub-domains (domain cassettes, DC) [5,7–9]; and homology in short sequence blocks found across the full gene (‘homology blocks’, HB) [7,9] or at positions of limited variability (PoLV) within the DBLα domain [10]. These different classification systems partially overlap [11,12]. Some *var* genetic groups have been consistently associated with severe disease, denoted here as ‘SM types’, namely, those with Group A-type upstream promoters (UpsA) [13], domain cassettes 8 and 13 [14], the REY motif at the PoLV2 position of the DBLα domain [15], and the presence of two cysteines (cys2) between the PolV3 and PolV4 positions in DBLα [13,16]. PfEMP1 host cell adhesion phenotypes ICAM-1, EPCR and rosetting have been mapped to expression of specific domains or domain cassettes thus providing a clear link between PfEMP1 diversity, adhesion phenotype and disease severity [4].

Antigenic properties of PfEMP1 are, by contrast, poorly understood. It is known that antibodies against *var* types associated with severe malaria, such as UpsA and DC8, are readily detected in young children living in malaria-endemic regions and develop before antibodies to other types [13,15,17–20]. In addition, VSAs from parasites found in younger children and those with severe disease are more frequently recognized by sera than those from older or more immune children: that is, they are more ‘immunologically common’ [2,13,15,21,22]. However, the antigenic properties that differentiate these types are not known. It has been hypothesised that some *var* types–those found in younger and sicker children—have immunological properties that differentially affect the quality and efficacy of the PfEMP1 antibody response [13]. Possible reasons for stronger antibody recognition of these types are that they have higher immunogenicity, elevated transcriptional levels, greater protein abundance on the red cell surface, stronger antigenic conservation, wider cross-reactivity or better ability to grow inside the host and thus become immunodominant. Some of these hypotheses are supported by recent studies [13,15,20,23]. Here, we describe the antigenic diversity of PfEMP1 in relation to severe vs. mild malaria in order to understand this further. To do so, we borrow a technique used in the study of immune cross-reactivity of influenza A viruses called ‘antigenic cartography’ [24,25] that is used to annually update flu vaccines in order to cover the ‘antigenic space’ of the circulating pathogen population [26]. Our overall aim is to lay the foundation for inferring the subset of antigens that might form a broadly protective PfEMP1-based vaccine.

## Results

### Serological and DBLα-tag antigen maps

**Fig 1:**
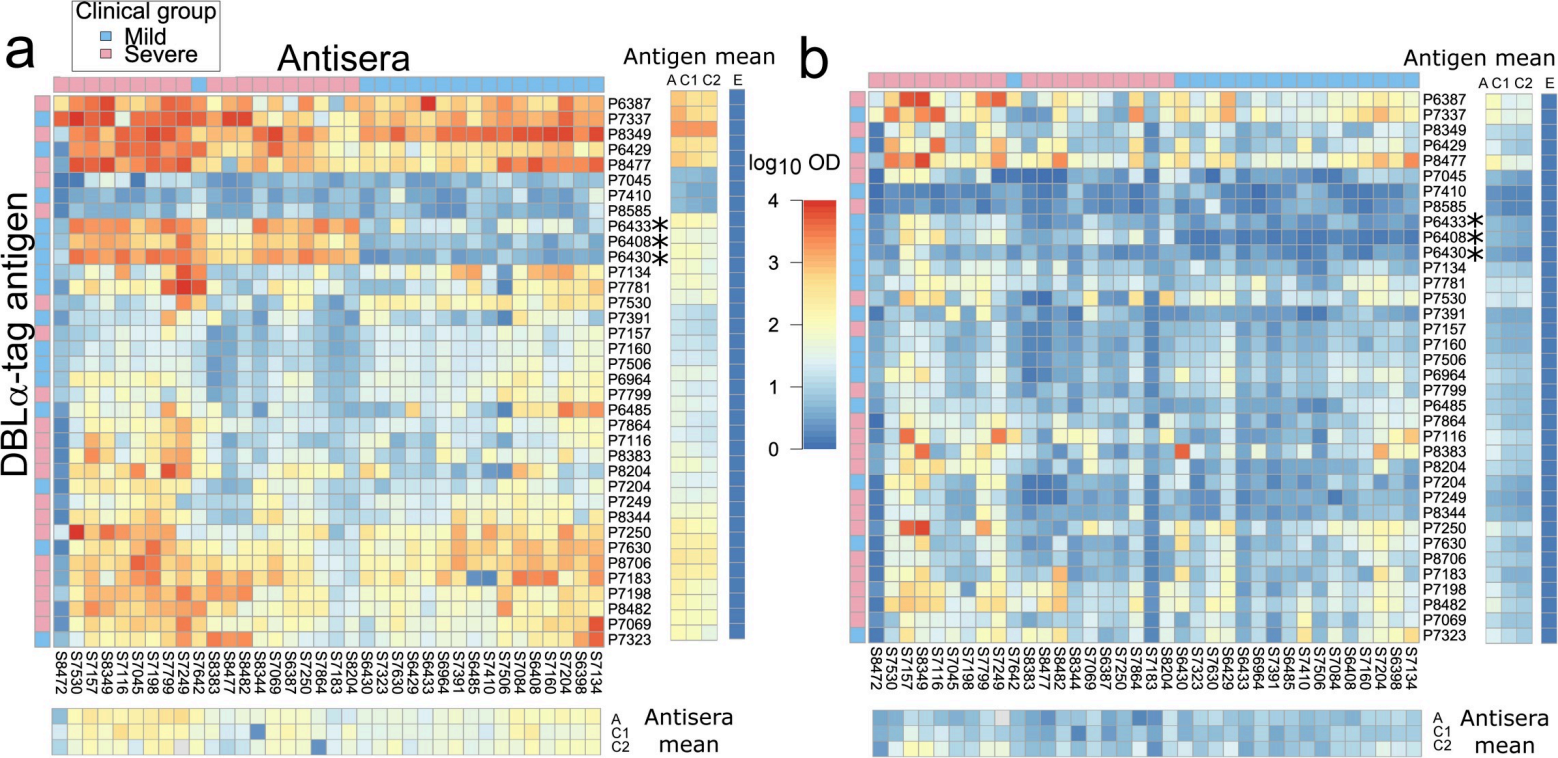
Sero-reactivity patterns of 36 plasma to 36 parasite DBLα-tag recombinant antigens. Square panels show strength of reactivity in log_10_ OD units (colour bar in centre) of pairwise combinations of 36 plasma (‘antisera’, columns) and 36 DBLα-tag antigens (rows) at the acute stage of infection for IgG (**a**) and IgM (**b**). Colour bars above and to the left of the heatmaps indicate clinical group (legend at top left). Right side panels show the mean strength of reactivity for the antisera or DBLα-tag antigen at the acute (A) and convalescent stages of the infection (C1 and C2, see S1 Fig). Corresponding means for plasma from eight Europeans are shown in the colour bar marked ‘E’: their 95% confidence intervals were below 0.20 log_10_ OD units for both IgG and IgM. Antisera and antigen labels indicate the patient number from which parasite material (isolate) and plasma were sourced, prefixed with S and P, respectively. Asterisks mark ‘indicator’ antigens, i.e., antigens that drove the topology of serological maps.

We measured sero-reactivity, both IgG and IgM, of anti-sera from 36 children with severe or mild malaria to a panel of recombinant proteins that represented part of the DBLα domain -the DBLα-tag—of dominantly expressed PfEMP1 types found in clinical isolates of 36 children (Fig 1, S1 Fig). 32 of the antigens derived from the 36 children that provided antisera. As expected given the endemicity of malaria in our study site in coastal Kenya, at the time of recruitment, children in our study had substantial prior history of malaria exposure, evident from their generally broad recognition and significantly higher sero-reactivity than unexposed control individuals to recombinant DBLα-tag proteins (Fig 1). Sero-reactivity of IgG with individual DBLα-tag proteins was generally higher than sero-reactivity of IgM at acute disease. This is most likely to be because IgG plasma antibodies reactive with individual DBLα-tags were generated during previous infections while IgM responses present induction of *de novo* responses from naïve B cells and, to a lesser extent, IgM memory B cells. We used analysis of variance to determine the principal factors affecting sero-reactivity: these revealed that both antisera and DBLα-tag antigens varied substantially in their average reactivity across antigens/sera, respectively explaining 39% and 12% of the total variation for IgG, and 13% and 7% for IgM under analysis Model 1 (Fig 1). These differences were maintained across timepoints (Fig 1, S1, S2 and S3 Figs). In addition, there was strong specificity of reactivity of individual anti-sera to individual DBLα-tag antigens: this explained a further 24% and 15% of the total variation in IgG and IgM reactivity, respectively, after excluding data from homologous (i.e., derived from the same child) DBLα-tag-antiserum pairs. These patterns of sero-reactivity were exploited to produce ‘serological maps’ that reflect shared patterns of reactivity of individual sera with all DBLα-tag antigens, and ‘antigen maps’ that reflect shared patterns of reactivity of individual DBLα-tags with all anti-sera. Distances between points in these maps correspond to differences in reactivity patterns between antigens or between sera, respectively.

**Fig 2:**
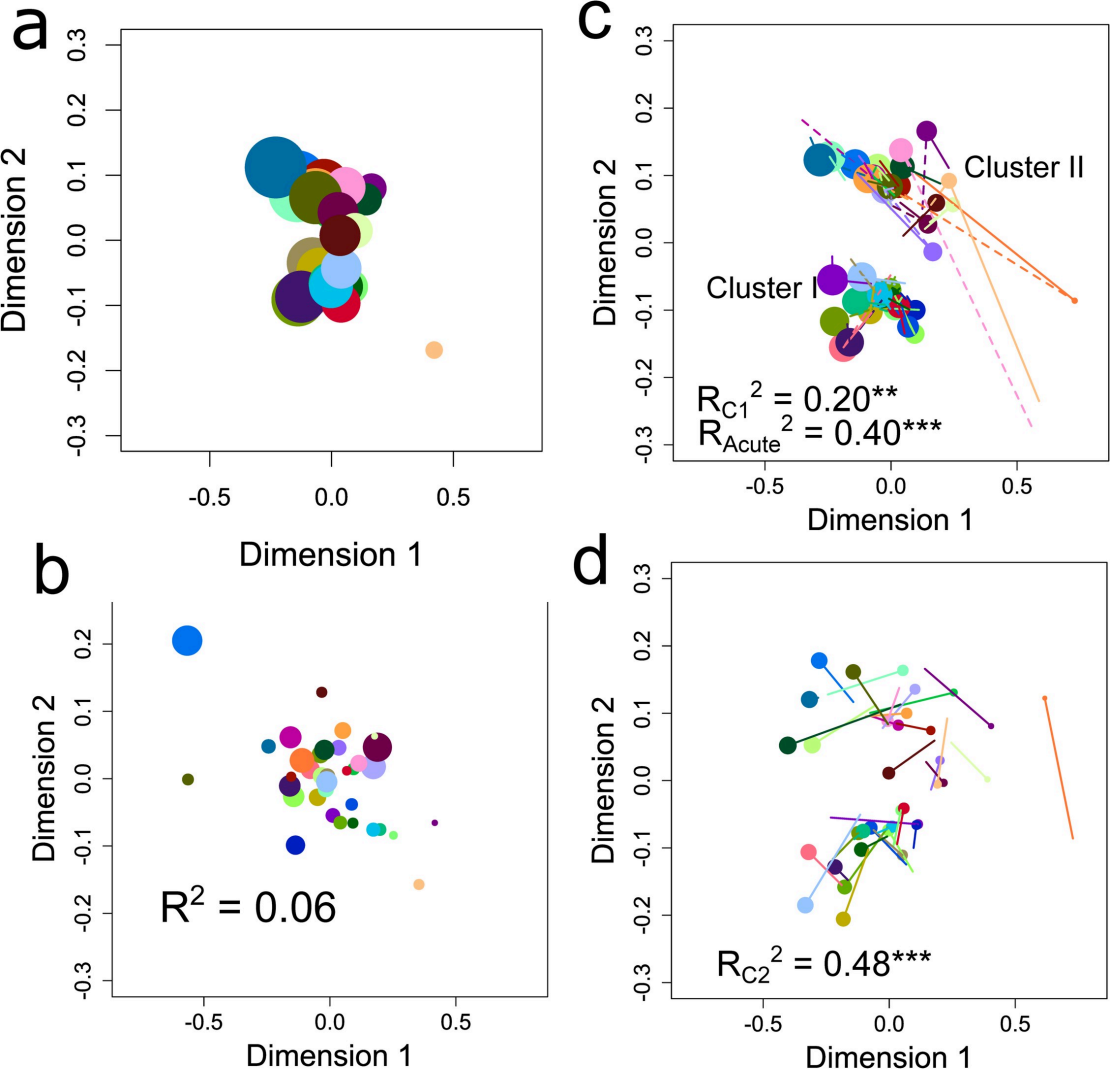
Antisera maps for IgG and IgM. Maps were constructed from data on **a**, IgG at the acute timepoint; **b**, IgG at timepoint C2 (the consensus antisera map); **b**, IgM at the acute timepoint; **d**, IgM at timepoint C2. In **c**, solid and dashed lines point to positions in the map for IgG at the acute (as in **a**) and C1 timepoints, respectively. In **c** and **d**, solid lines point to positions in the consensus antisera map shown by the points in **c**. Symbol size is proportional to the antisera’s average log_10_ OD value at the timepoint indicated. R^2^ values represent goodness-of-fit to the consensus map with subscripts indicating the timepoint of the map being compared and asterisks indicating significance (***, P < 0.001; **, P < 0.01).

Serological maps revealed two distinct clusters which we denote serological Clusters I and II hereon. For IgG, these serological clusters were apparent at the acute stage (Fig 2A) and remained stable throughout the convalescent period (Fig 2B, S1 & S4 Figs). By contrast, for IgM, serological clusters were not evident at the acute stage (Fig 2C) but, as convalescence progressed, despite the overall low sero-reactivity compared to IgG, two clusters emerged which were the same as those for IgG (Fig 2D, S5 Fig). Serological clusters were still evident after pre-adjusting sero-reactivity data for mean antisera reactivity (S4 and S5 Figs vs. Fig 2) and thus were not generated by systematic differences in mean reactivity of sera. Instead, they were driven by the highly specific nature of serum-DBLα-tag reactivities: this was most obvious for serological Cluster II antisera which reacted strongly with DBLα-tag antigens P6433, P6408 and P6430 while serological Cluster I sera did not (Fig 1, S1 Fig).

DBLα-tag antigen maps, by contrast, did not show strong clustering at any timepoint for either IgG or IgM (S6, S7 and S8 Figs). Instead, distances between DBLα-tag antigens were principally a function of differences in a given DBLα-tag antigen’s mean reactivity (S6 Fig). Nonetheless, DBLα-tag antigens grouped into five groups (denoted DBLα-tag antigen Clusters III to VII) using a combination of hierarchical clustering (S7 Fig) and the proportion of antisera strongly recognized (log_10_ OD > 2) as follows: Antigen Cluster III, ‘high reactivity’ with >75% anti-sera reactive with these DBLα-tags; Antigen Cluster IV, ‘high specificity’ with 40–60% of anti-sera strongly reactive and the remaining anti-sera with low reactivity; Antigen Cluster V, ‘medium specificity and reactivity’ with medium reactivity of anti-sera to a broad set of DBLα-tag antigens (19–64%) that were largely non-overlapping with the highly reactive antigens that defined Cluster IV; Antigen Cluster VI with ‘low reactivity’ with <25% of anti-sera reactive; and Antigen Cluster VII with ‘zero reactivity’ with 0% of anti-sera reactive. Antigen clustering weakened upon pre-adjusting for antigen mean reactivity (S7 and S8 Figs vs. S1 Fig). Antigenic clusters did not significantly associate with serological clusters (S6 Fig, P = 0.20 by chi-squared test of association on 4 d.f.).

**Fig 3:**
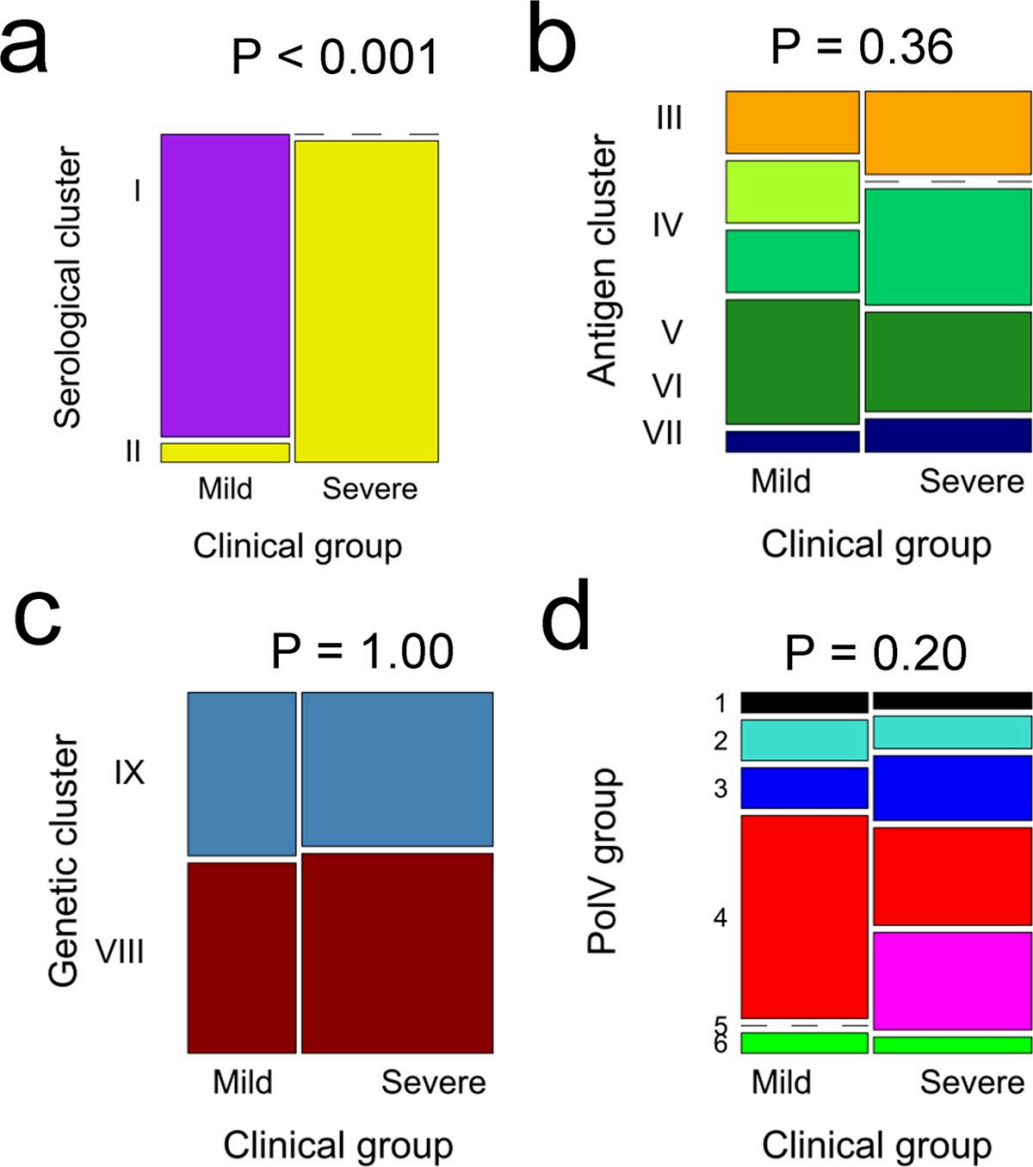
Associations between disease severity and clusters. Frequency distributions of clinical group (x-axis, disease severity) by **a**, serological cluster; **b**, antigen cluster; **c**, genetic cluster; **d**, PoLV group. Significance (P-values) for chi-squared tests of associations between clinical group and clusters are shown at the top of each panel.

Serological clusters almost perfectly aligned to clinical group and thus disease severity: whereas all serological Cluster I sera derived from patients with mild malaria attending the hospital outpatients department (n = 16), all but one (n = 19) of the Cluster II sera derived from patients admitted to the hospital wards (Fig 3A, P < 0.001 by chi-squared test on 1 d.f.). Since host age, parasite density at the time of sampling and average reactivity of the serum did not differ significantly between clinical groups (Table 1, P > 0.05 by t-tests), the association between serological clusters and disease is unlikely to have arisen from different levels of prior exposure in the two clinical groups. There was a significant effect of clinical group on date of sampling because all six plasma samples collected in 2008 were sourced from patients admitted to the hospital (Table 1). However, since the association between serological clusters and disease severity remained highly significant (P < 0.001) after excluding data from the 2008 samples, this confounding was not responsible for the association, as might be the case, for example, if the frequency of severe malaria-causing genotypes in the population changed from year to year.

**Table 1:** Associations between clinical parameters, serological responses, parasite genetic factors and clusters found in serological and genetic diversity maps.

		Clinical group median (range)		P-value^b^			
	ups group & DC^a^	Mild disease (n = 16)	Severe disease (n = 20)	Clinical group	Serological cluster	Antigen cluster	Genetic cluster
**Host and infection parameters**							
Parasite density (p/μl)		170,000 (13,440–680,000)	201,600 (18,414–1,680,000)	0.2403	0.1239	0.2442	0.8996
Age (mo)		45 (9–83)	40 (4–73)	0.2928	0.9841	0.7870	0.6981
Date		Jun 2006 (Jul 2005–Jan 2007)	Jan 2007 (Jul 2005 –Jul 2008)	**0.0002**	**0.0003**	0.3310	0.7929
Mean IgG antisera reactivity^c^		1.82 (1.52–2.23)	1.93 (1.05–2.40)	0.8693	0.9551	0.2579	0.4592
Mean IgG antigen reactivity^d^		1.77 (0.60–2.83)	1.81 (0.66–3.34)	0.5894	0.2708	**<0.0001**	0.6522
Mean IgM antisera reactivity		0.90 (0.63–1.14)	0.91 (0.43–1.60)	0.9333	0.9923	0.1647	0.1154
Mean IgM antigen reactivity		0.83 (0.29–1.75)	0.96 (0.29–1.84)	0.3011	**0.0187** (I<II)^e^	**0.0019**	0.4959
**Parasite genetic factors**							
Global frequency^f^		0 (0–31)	1 (0–50+)	0.3107	0.4279	0.9894	0.1511
Homology Block 1^g^		29.8 (10.5–34.5)	22.9 (0–33.1)	**0.0031**	**0.0005**	0.5012	0.4995
Homology Block 54		0 (0–18.6)	11.7 (0–20.5)	0.0717	0.0716	0.3087	0.0705
Homology Block 64		6.3 (0–18.2)	0 (0–16.9)	**0.0443**	0.0883	0.6857	0.0496
Homology Block 36		35.0 (0–58.1)	37.2 (0–50.4)	0.7212	0.8615	0.3659	**<0.0001**
Homology Block 60		5.1 (0–38.6)	0 (0–46.6)	0.8475	0.8853	0.3303	**<0.0001**
DBLa_CIDRa^h^	B (DC8)	0.2 (0–11.1)	2.7 (0–46.6)	**0.0430**	0.1269	0.3583	0.4571
DBLg4/6	B (DC8)	0.1 (0–19.0)	7.4 (0–33.7)	**0.0064**	**0.0116**	0.4568	0.6836
CIDRa1.1	B (DC8)	0.1 (0–661)	11.6 (0.1–115)	**0.0073**	**0.0246**	0.4143	0.9236
DBLb12 and DBLb3/5	A/B (DC8)	0.4 (0–64.7)	12.7 (0.1–79.0)	**0.0058**	**0.0264**	0.2935	0.4642
DBLz4	B (DC9)	1.7 (0–14.9)	4.2 (0–82.7)	0.8533	0.7234	0.4496	0.3562
CIDRa1.4	A (DC13)	0.0 (0–2.9)	0.3 (0–50.7)	**0.0025**	**0.0098**	0.6315	0.8664
DBLa2/a1.1/2/4/7	A	3.0 (0.3–101)	41.7 (2.5–134)	**0.0109**	**0.0357**	0.2866	0.3085
CIDRa1.6	A	0.0 (0–17.2)	0.1 (0–48.3)	0.3678	0.5028	0.6319	0.0629
DBLa1 not var3	A	3.4 (0.1–128)	40.8 (0.8–258)	**0.0058**	**0.0163**	0.1801	0.2449
b1	B	2.0 (0–29.5)	10.2 (0.8–157)	0.0590	**0.0475**	0.5151	0.9812
c2	C	0.7 (0–2.8)	2.7 (0.1–19.8)	**0.0045**	**0.0121**	0.6856	0.3666

a. Classification based on upstream promoter (ups) and domain cassette (DC, in parentheses).

b. P-values (P < 0.05 in bold) for t-tests of the effects of clinical group, serological cluster, antigen and genetic cluster (columns, assessed under Models 7, 3, 5 and 6, respectively) on host and infection factors (rows, top section) and for Wilcoxon rank tests for parasite genetic factors (lower section).

c. Mean OD for antibodies belonging to the group indicated in the table column.

d. Mean OD for antigens belonging to the group indicated in the table column.

e. The mean for serological Cluster I was lower than for Cluster II.

f. Number of isolates in the *var* gene database [30] with >95% DNA sequence identity to the full DBLα-tag. ‘50+’ denotes >50.

g. Homology block as defined in ref. [9]. Values are scores from BLAST analyses in the *var* gene domain database [31].

h. Primer name for measuring transcript levels as defined in ref. [14].

DBLα-tag antigen clusters did not significantly associate with clinical group (P = 0.36 by chi-squared test on 4 d.f., Fig 3B). However, all three of the DBLα-tag antigens with very high specificity, reacting strongly to approximately half the plasma (antigen Cluster IV), derived from patients with mild disease who had serological Cluster I profiles. DBLα-tag antigen cluster was not significantly associated with other host infection parameters (Table 1).

### Effects of heterology of individual antigen-antiserum pairs

**Fig 4:**
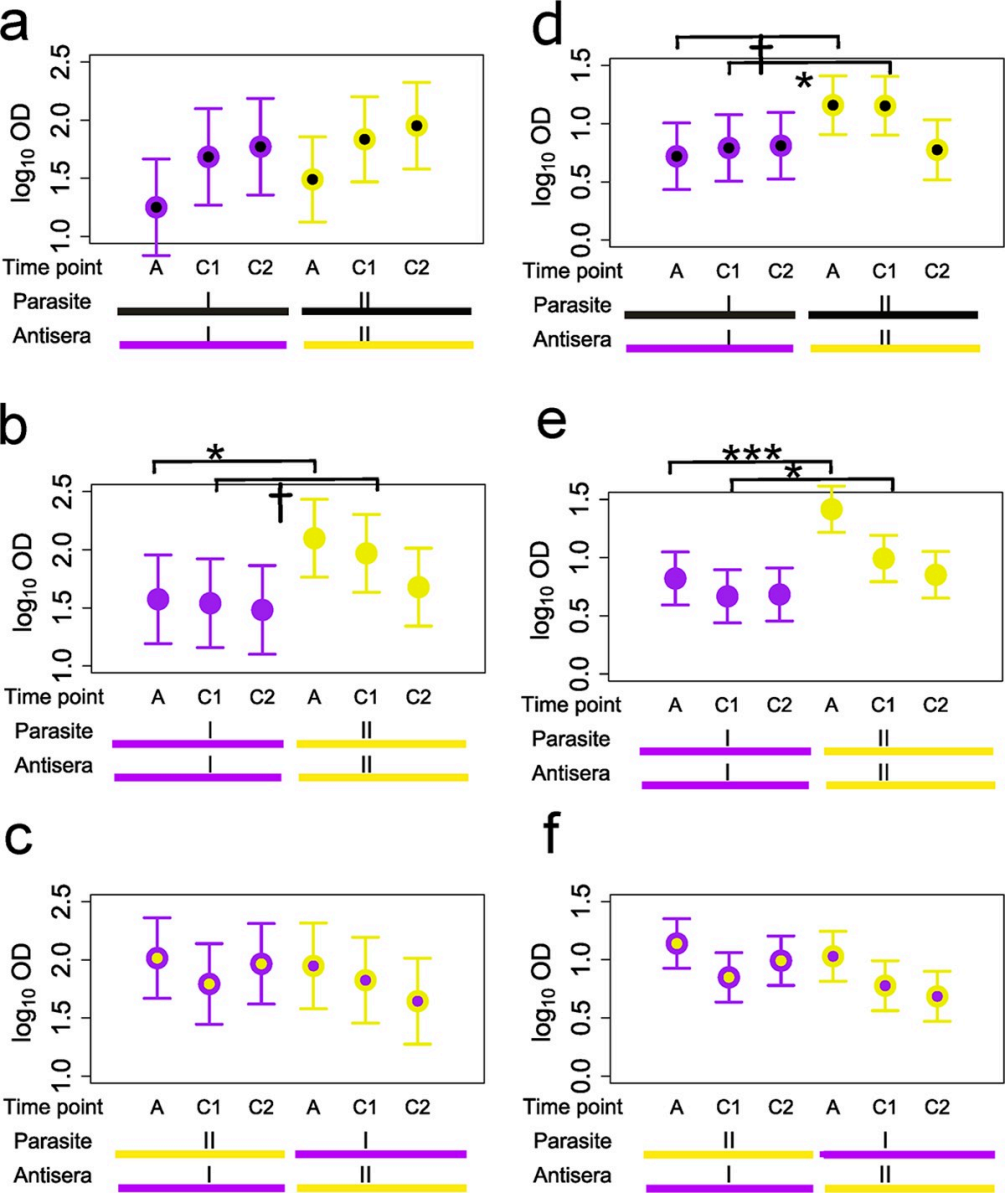
Effects of heterology. Each panel shows least-squares means (circles) with 95% confidence intervals (vertical bars) of IgG (**a**, **b**, **c**) and IgM (**d**, **e**, **f**) sero-reactivity for DBLα-tag antigen-antisera pairs split by timepoint and serological cluster (Antisera I and II on x-axis). In **a** and **d**, data are from antigen-antisera pairs that derive from the same host, (i.e., are homologous). In **b** and **e**, data are from antigen-antisera pairs that are heterologous with respect to individual antigens/antisera but which derive from hosts in the same serological cluster. In **c** and **f**, antigen-antisera pairs are heterologous with respect to both individual antigens/antisera and serological cluster. Colour of outer circles and lower horizontal bars below the x-axis indicate serological cluster; colour of inner circles and upper horizonal bars indicates serological cluster of the host from which the DBLα-tag antigen derived. Black inner circles and upper black horizontal bars indicate homologous antigen-antisera combinations. P-values of statistical tests for the effect of serological cluster at each timepoint are shown in black text with horizontal brackets. n.s., P > 0.05; ***, P< 0.001.

At the acute stage of infection, IgG sero-reactivity of homologous antigen-antiserum pairs (i.e., both the plasma and parasite isolate from which the dominant DBLα-tag was cloned were sourced from the same child) was significantly lower than for heterologous antigen-antiserum pairs (P < 0.001 under Model 2, Fig 4A vs. 4B and 4C). This effect disappeared in the convalescent phase during which antibodies to homologous DBLα-tags significantly increased (P < 0.001 fitting timepoint as a linear covariate) while those to heterologous DBLα-tags significantly declined (see below). These findings are consistent with those from previous studies showing that children are more likely to become infected with PfEMP1 antigenic types not encountered previously, and that they subsequently mount antibody responses that are primarily specific to the infecting antigenic type [2,27–29].

### Serological clusters differ in their quality of immune response

There were no differences between serological Cluster I and II in the homologous IgG response (P > 0.05 at all timepoints under Model 4, Fig 4A). By contrast, Cluster I and II antisera responded differently to heterologous DBLα-tag antigens. Acute stage plasma belonging to serological Cluster I had lower IgG reactivity to heterologous DBLα-tag antigens from children with the same serological cluster than to antigens from the opposite cluster (Fig 4B vs. 4C, P < 0.01 and P < 0.05 at the A and C2 timepoints under Model 3), similar to the heterology effect for individual antigen-antisera pairs. By contrast, serological Cluster II had similar or higher IgG sero-reactivity to DBLα-tags from children with the same serological cluster than to antigens from the opposite cluster (P = 0.04 for timepoint C3 and P > 0.05 at the A and C2 timepoints under Model 3, Fig 4B vs. 4C). Serological Cluster II profiles further differed from serological Cluster I profiles in that–with the exception of responses to homologous antigens—antisera levels declined significantly during convalescence (Fig 4B and 4C, P < 0.001 for Cluster II vs. P > 0.05 for Cluster I fitting timepoint as a linear covariate in Model 3). Serological cluster and cluster heterology effects for IgM patterns were qualitatively similar, though weaker, to those for IgG (Fig 4D, 4E and 4F).

Combined, these results suggest that children develop one of just two qualitatively distinct serological profiles with respect to the DBLα-tag antigen which align with disease outcome of the infection. Serological Cluster I profiles appear to be broadly reactive, long-lasting and, while less effective in protecting against infection with non-SM than SM types, nonetheless are effective in preventing progression to severe disease. Serological Cluster II profiles, by contrast, while generally stronger at the time of acute infection, appear to be narrowly specific, transient, non-protective against infection with all types—both SM and non-SM—and ineffective in preventing progression to severe disease.

### Genetic diversity in DBLα-tag antigens and correspondence with serological and antigenic diversity

**Fig 5:**
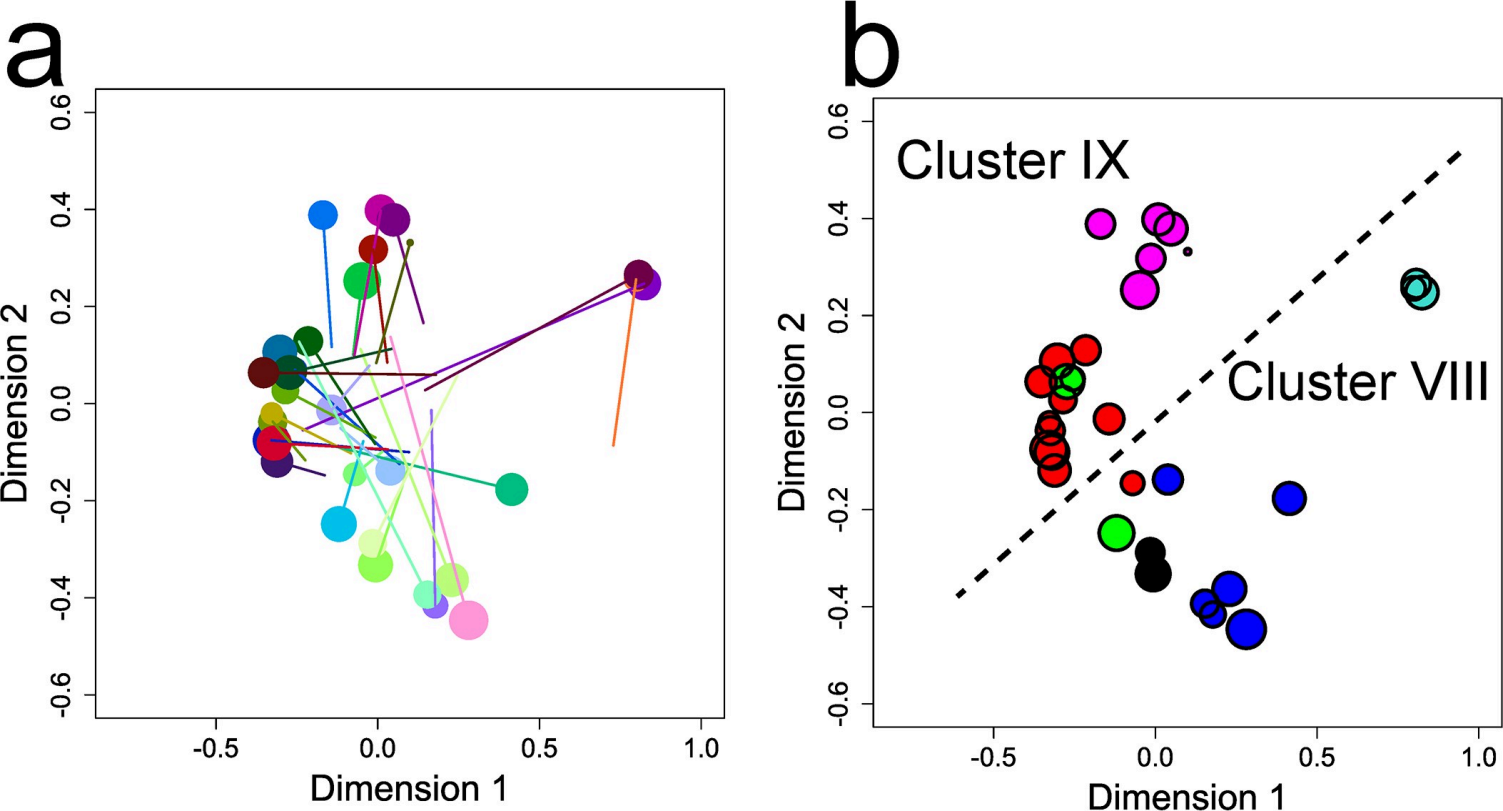
Genetic maps. **a**, Genetic map (points) with reference (lines) to the consensus antisera map (IgG at C2). Goodness-of-fit was not significant (R^2^ = 0.07, P = 0.25). **b**, Genetic map coloured by PoLV groups. Point size is as for the consensus antisera map (see Fig 2).

Genetic maps based on the full amino acid sequence of the DBLα-tag region of PfEMP1, like serological maps, revealed two genetic clusters (denoted Genetic Clusters VIII and IX) (Fig 5). These corresponded to the 2-cysteine vs. not-2-cysteine major genetic groupings described previously [10] (Fig 5B, P < 0.001 by chi-squared test). Genetic cluster and PoLV group did not significantly associate with clinical group (Fig 3C, P = 1.00 and P = 0.20, respectively), serological cluster (P = 1.00 and P = 0.13, respectively), or DBLα-tag antigen cluster (P = 0.91 and P = 0.35, respectively) or any of the host and infection variables described above (Table 1).

**Fig 6:**
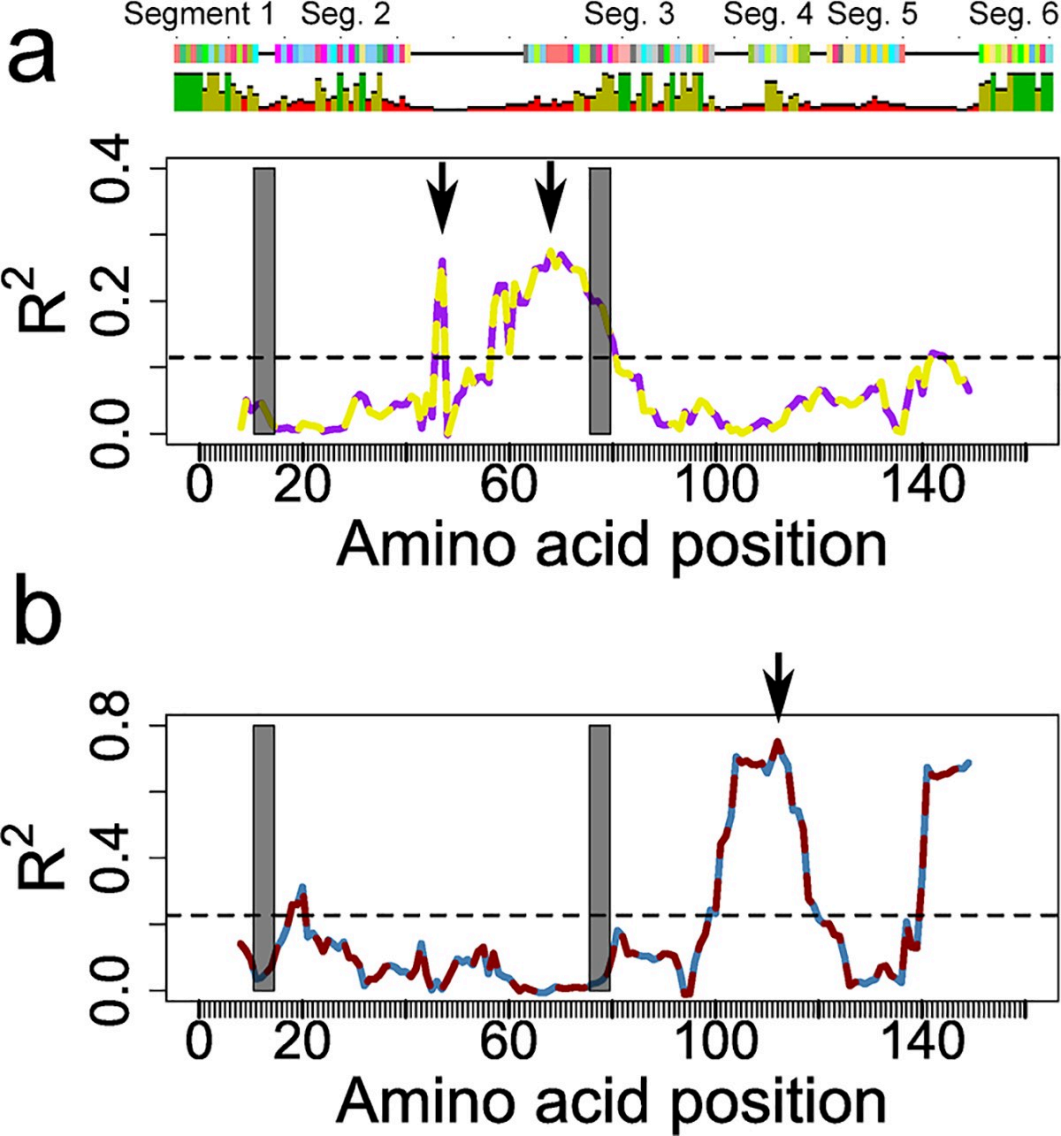
Sliding window analysis for regions of sequence that predict antigenic and genetic clusters. Correspondence (y-axis, measured by R^2^) between genetic sub-maps built from sequence similarity in 14-residue windows (x-axis, midpoint of window) and **a**, serological clusters; and **b**, genetic clusters. Positions (window midpoints) that best define serological and genetic clusters, respectively, are shown with vertical arrows. Horizontal dashed lines in **a** and **b** indicate the P = 0.05 significance level for R^2^ from permutation tests. PoLV1 and PoLV2 positions are indicated by grey boxes. The consensus sequence is shown at the top of panel **a**.

**Fig 7:**
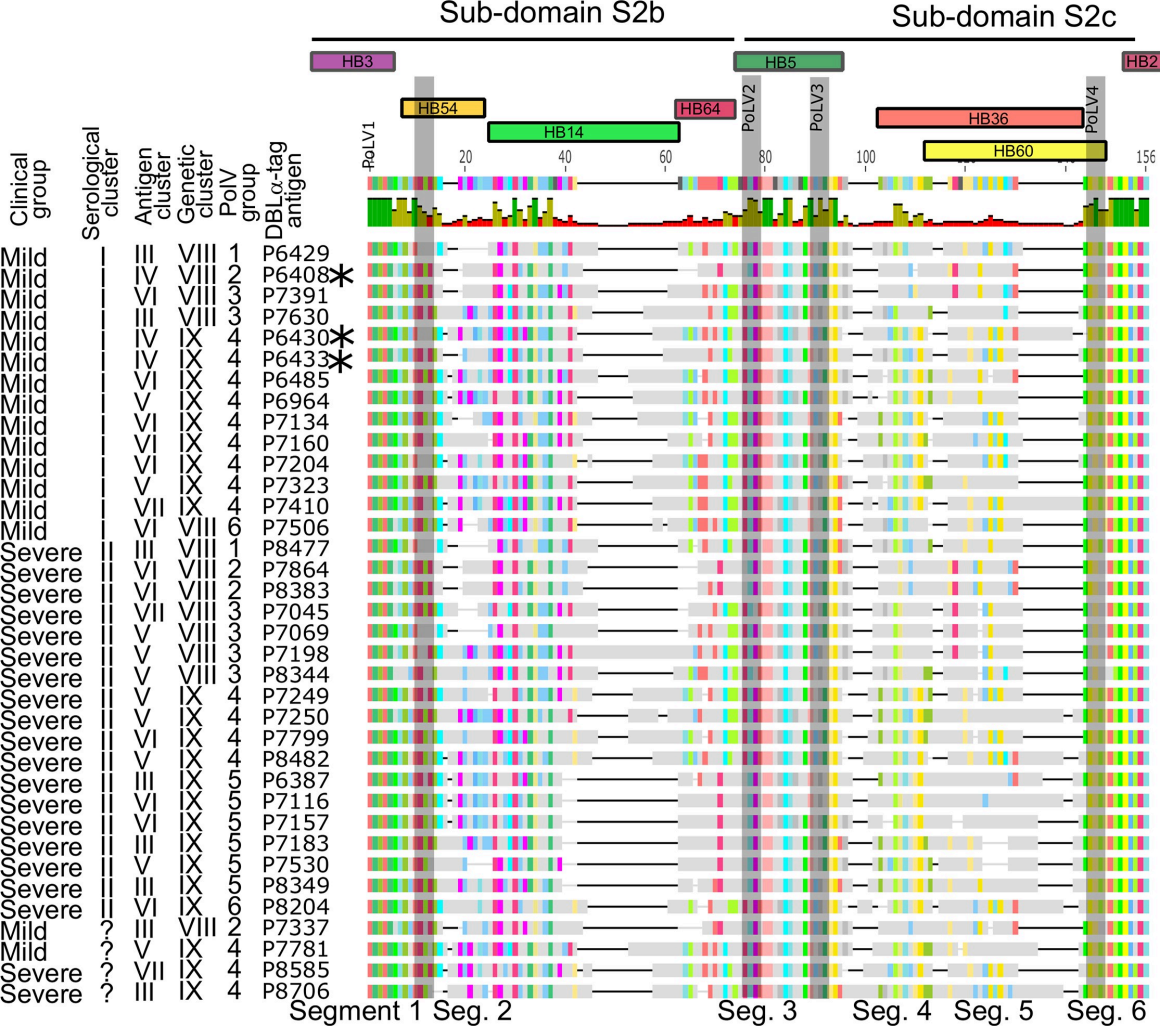
Amino acid sequence alignment of the DBLα-tag recombinant proteins. Coloured residues indicate identity with the consensus sequence which is shown at the top; grey residues indicate differences from the consensus. Dashes indicate non-presence of the consensus residue. The sequence divides into six segments of moderate homology (black text below figure) and into two sub-domains, S2b and S2c as defined in ref. [9] (black horizontal lines at top). Positions (window midpoints) that best define serological and genetic clusters, respectively, are shown with vertical arrows, corresponding to those shown in Fig 6A and 6B, respectively. Parasite isolates, their clinical group, cluster membership and PoLV groups are indicated in black text to the left of the figure. Coloured blocks at the top of the figure show homology blocks (HB) as defined by Rask et al. (2010). The most highly conserved HBs across the global population are shown at the top: below these are less conserved HBs that showed significant associations with disease severity, serological cluster (HB14, HB54 and HB64) and genetic cluster (HB36 and HB60) in this study (Table 1).

Sliding window analysis of genetic maps based on 14 amino acid sub-regions in relation to serological, DBLα-tag antigenic and genetic clusters indicated that serological clustering was principally driven by genetic diversity between Segments 2 and 3 at the N-terminal portion of the DBLα-tag ending with PoLV2 (Fig 6A and S9 Fig). This region comprises almost complete S2b and S2c sub-domains of DBLα domains as defined by Rask et al. (2010) [9]. High concordance between serological clustering and genetic diversity in this region was driven by the fact that all but one (P6408) of the nine antigens that contained a REY motif at the PoLV2 position (PoLV Groups 2 and 5) derived from patients with serological Cluster II profiles, while only one REY type was found in patients with serological Cluster I profiles (Fig 7, S9B and S9E Fig). Previous evidence suggests that parasites expressing PoLV/Cys Group 2 [10] and Group 5 [32] are likely to form rosettes, a cytoadhesion phenotype strongly implicated in severe disease [33–35]. This, together with our findings, suggests that epitopes in the S2b sub-domain of DBLα REY sequence type, or elsewhere in the PfEMP1 protein but in strong linkage disequilibrium with the latter, are the target of antibodies that are associated with a milder course of disease.

Genetic clustering was strongly associated with sequence diversity in Segments 4 and 5 at the 3’ end of the S2c sub-domain of DBLα, consistent with location of the cysteines that distinguish two major genetic groups under the PoLV/Cys classification system [10] (Fig 6B).

We explored other genetic aspects of the DBLα domain for their relationship with disease and serological clusters. Recognition scores for homology blocks HB14 and HB64 were significantly elevated in DBLα-tags derived from parasites from patients with mild disease and serological Cluster I profiles, while those of HB54 were elevated in DBLα-tags derived from parasites from patients with severe disease and serological Cluster II profiles (Table 1). These three homology blocks span Segments 1, 2 and 3 of the DBLα domain (Fig 7), thus corroborating the strong association between serological cluster and genetic diversity in the S2b sub-domain described above.

Recognition scores of HB60 and HB36 were significantly positively associated with Genetic Clusters VIII and IX, respectively (Table 1), consistent with their location in Segments 4 and 5 in which variation in the number of cysteines that delineate these clusters occurs (Fig 7).

### Genetic features outside the DBLα-tag

The analysis above focused on sero-reactivity of plasma to the DBLα-tags of the dominant expressed PfEMP1 in clinical isolates. We next addressed whether underlying genetic characteristics of the full length PfEMP1 protein in the entire parasite population of a given child, such as expression of specific domain cassettes or upstream promoter sequences were associated with serological or antigenic clusters. We found that expression levels of domain cassettes DC8 and DC13 were significantly positively associated with severe disease and serological Cluster II or both (Table 1), consistent with previous studies [14,20]. Expression levels for three out of four other markers of *var* subtypes associated with severe disease in previous studies were significantly associated with disease in this study (Table 1): two of these markers define DBLα sub-types that belong to the upstream promoter Group A (DBLa2/a1.1/2/4/7 and DBLa1). Surprisingly, a generic group C marker that has been previously associated with mild disease was also elevated in children admitted to hospital: however, expression levels of Group C upstream promoter regions were low in all groups (Table 1).

Global population frequency of the parasite’s *var* genes, as judged by number of highly similar sequences to the full DBLα–tag in the global *var* gene sequence database, did not associate with disease severity or clusters (Table 1). Thus, the association between disease severity and antigenic profile was not explained by the rarity of, and hence degree of prior exposure to the *var* gene products of the infecting isolate.

Overall, our genetic analysis of the parasites in this study confirms the conclusion from previous studies that a subset of genetically defined PfEMP1 types–those with DC8, two cysteines, and Group 2 and Group 5 REY types—are strongly predictive of severe disease. By introducing serology into analysis of this relationship, we have shown that protection against these SM types may depend on serological responses to epitopes in the S2b sub-domain of DBLα, or epitopes elsewhere in the PfEMP1 protein that are in strong linkage disequilibrium with these. By contrast, protection was not associated with putative epitopes in the S2c sub-domain which delineates the two major genetic groups by its number of cysteines, Cys2 and non-Cys2.

## Discussion

This study has revealed that, despite different histories of malaria and diversity of antigenic types, serological responses to PfEMP1 in children fall into one of just two qualitatively different patterns which strongly associate with disease severity. In most children, serological responses to PfEMP1 (here evaluated using recombinant DBLα-tags representing the dominantly expressed PfEMP1) are broadly reactive and long-lasting, but in some children–those that succumb to severe disease—responses are narrower and short-lived. This conclusion, based on the similarity of serological profiles between children to a set of related antigens rather than on the frequency of responses to individual antigens–as in most immuno-epidemiological studies—departs from the traditional view that protection against malarial disease is acquired in a piecemeal fashion through acquisition of specific antibodies to each of PfEMP1’s many antigenic types upon repeated exposure [36,37]. It shifts the focus in understanding severe malaria away from individual antigenic types towards the overall quality of the antibody response.

As in many previous studies [13,15,17–20], we observed that PfEMP1 (here represented by DBLα-tags) from parasites that caused infections which progressed to severe disease were generally better recognized by plasma than those from infections resulting in mild disease (Figs 1 and 4). How does this reconcile with our proposal that patients with severe disease mount serological responses that are less effective and less persistent? Although *var* genes are very heterogenous in sequence within and between genomes, they share sequence similarities that define domains, homology blocks and binding-sites for endothelial receptors, as also reflected in short shared stretches of sequence similarities within the DBLα-tag region [38]. These sites could be targets of cross-reactive serological responses. Cross-reactive antibody responses can be short-lived when the corresponding antibodies originate from memory B cells that differentiate into short-lived plasma cells, or long-lived when antibodies originate from long-lived plasma cells. Our data suggest that children with mild disease maintain stable antibody responses to heterologous DBLα-tag antigens, with levels generally higher to DBLα-tag antigens derived from parasites of children with severe malaria, while children with severe disease have similar levels of serological responses to heterologous DBLα-tag antigens of all parasite types which drop significantly during convalescence. Given that memory B cells tend to leave germinal centres earlier than long-lived plasma cells during affinity maturation [39], it seems likely that the short-lived serological Cluster II IgG responses originated from short-lived plasma cells that had lower affinity for heterologous DBLα-tags, and that these qualitative differences contributed to the association of serological profiles with disease severity that we observed here.

Remarkably, serological profiles for IgM and IgG were similar at the convalescent stage. IgM memory B cells contribute—and sometimes are the earliest responders [40]–to the rapid boosting of antibody levels during re-infection. However, differentiation of naïve B cells in response to new antigenic variants increases levels of plasma IgM memory B cells too. Although the source of plasma antibodies to the different DBLα-tag antigens has to be further investigated, we thus hypothesise that the different sources and quality of memory B cells may underlie the reason for why children that have developed Cluster I serological profiles in response to previous infections are protected from severe disease, even when they become infected with SM types, while children with Cluster II profiles are not. We further note that the decoupling of the roles of antigen and serological responses in severe disease under this hypothesis contrasts with that for establishment of an infection which clearly depends on highly specific reactivity to individual antigenic types.

Although severe disease is clearly linked with PfEMP1 type, such as expression of DC13, DC8 or group A PfEMP1 associated with rosetting, expression of these SM-PfEMP1 types is not sufficient for the development of severe disease since they are also expressed during first infections, whether or not the child develops severe malaria, and because children readily induce antibodies against SM-PfEMP1 [14]. Our data suggests that this gap is filled by some qualitative feature of the host’s immune response that causes failure to develop an adequate anti-SM-PfEMP1 response. Possible reasons for this include host-derived factors that influence the general quality of the immune response such as haemoglobinopathies which alter display of PfEMP1 on the red cell surface [41]; parasite-induced epigenetic modification of immune cell function [42]; PfEMP1-mediated inflammation resulting in dysregulation of T- and B cell function [43] or antibody-dependent cellular cytotoxic function [44]; and RIFIN-mediated binding to LILRB1 expressed on B cells [45]. Clearly, underlying host genetic factors and cellular events that result in distinct serological profiles between children with mild vs. severe malaria disease require further investigation.

Alternatively, the failure may be directly attributable to the parasite. Theoretical modelling of within-host infection dynamics has led to the hypothesis that in order to produce sequential expression of individual *var* genes during the course of an infection, the parasite must induce the immune system to produce long-lasting, cross-reactive antibodies as well as transient, highly specific antibodies to the current numerically dominant variant [46]. Empirical studies have shown that, in patients with severe disease, *var* gene expression is dysregulated, leading to the appearance of multiple *var* types simultaneously instead of sequentially: this has been linked to down-regulation of parasite histone deacetylase PfSIR2A, an epigenetic silencer of *var* gene expression [20,47]. The latter two observations, together with our results here, support a model in which hosts mount one of two types of immune response to PfEMP1 –one (Cluster I-like) in which *var* gene expression is well-regulated, this with assistance from long-lasting antibody seroreactivity, and which culminates in control of severe disease; and the other (Cluster II-like) in which many *var*s are expressed simultaneously, generating an antibody repertoire that is broader but less effective, presumably as a consequence of generating fewer antibodies to each type and which thus gives the appearance of transience.

Such a model must include an antigen-antiserum specific component, however, since our conclusion that protected children mount broader and longer serological responses than unprotected children does not sufficiently explain the serological reactivity patterns in our data. We found that it was not the overall quantity of plasma antibody, but instead the highly specific nature of the immune profiles which delineated serological Cluster I from Cluster II and mild from severe disease patients. The serological specificity that drove these differences was particularly obvious among a small subset of DBLα-tag antigens (P6433, P6408 and P6430, Fig 1, S7 Fig). Perhaps surprisingly, these ‘indicator’ antigens were only recognized by children with severe disease and all of them derived from children with mild disease (S1 Fig). We therefore interpret strong responses to this subset of antigens in children with severe malaria as an indication of, rather than the cause of, a defective immune response.

A variant-specific basis for the relationship between serological antibody profile and disease outcome is further supported by the results of our analysis relating distinct serological profiles to genetic variation within PfEMP1. We mapped diversity in serological responses to genetic diversity in HB14, a short region of semi-conserved sequence in the S2b sub-domain of PfEMP1’s DBLα domain. HB14 spans a hypervariable loop (Fig 6 & S9 Fig) between two alpha helices that form the core structure of this sub-domain: these are marked by conserved sequences HB3 at the 5’ end and HB5 at the 3’ end. HB5 is believed to be frequently exposed on the surface of PfEMP1 [9]: the adjacent hypervariable region marked by HB14 may also be frequently exposed to the host’s immune system. Just downstream from HB14 and marking the beginning of HB5 is a short motif–REY or RED. REY defines genetic sub-groups (PoLV Groups 2 and 5) which have previously been reported to be associated with severe malaria, particularly rosetting [32]. REY-type HB5 tend to have shorter segments in the HB14 region (Fig 7). Sero-reactivities against REY-containing DBLα-tag sequences are acquired early in life in children living in Papua New Guinea [48]. Taken together, these findings suggest that the length and/or diversity of the hypervariable loop exposed on the surface of PfEMP1, as defined by HB14, might provoke antibody responses that qualitatively differ in their efficacy in controlling progression to severe disease.

Since we did not sequence the full length of the *var* genes, we could not determine the complete set of genetic variants that best predict serological profile and how these relate to Group A, DC8 and DC13 *var* types that generate severe malaria. Given the genetic structure of *var* genes, it is likely that HB14 and HB5 in the DBLα domain are in linkage disequilibrium with cassette types in other domains. A much larger study is required to identify all the potential epitope sites in PfEMP1 that could explain the dichotomous serological profiles that we have revealed here. Such a study would constitute a significant step towards the development of a broadly effective PfEMP1-based malaria vaccine. Unlike vaccines for other highly genetically diverse pathogens such as influenza A, in which antigen components are selected to cover the full antigenic diversity of the currently circulating population, and which require updating for each global hemisphere every year [25,26], the challenge will be to define PfEMP1 types that elicit the long-lasting, protective version of the antibody repertoire, as found in most children in malaria-endemic areas, and which we have begun to define here.

## Materials and methods

### Ethics statement

Children were recruited into this study after written informed consent from their parents or guardians. Ethical permission for the study was granted by the National Ethical Review Committees of Kenya (Kenya Medical Research Institute protocol SSC1131) and the Oxford Tropical Research Ethics Committee (OXTREC protocol no. 30–06).

### Study population

Thirty-six children under the age of 5 years presenting with acute malaria at the Kilifi County Hospital, Kilifi, Kenya each donated a venous blood sample at recruitment (Acute, A), at 4 weeks (Convalescence 1, C1) and at 16 weeks (C2) after the acute malaria episode. Children were either admitted to the hospital ward according to pre-established criteria for severe malaria (Blantyre coma score <5, Hb<5g/dl or respiratory distress [49]) or moderate malaria (admitted to the ward but without any of these severe malaria syndromes): otherwise they were diagnosed as ‘mild malaria’, treated with anti-malarial drugs at the Outpatient Department (OPD) and sent home. Plasma and parasite material were collected upon recruitment and separated and stored using standard procedures [50].

### Antigen preparation

The dominant expressed *var* gene in each isolate at the time of recruitment was identified based on the DBLα-tag as described previously [50,51]. For 36 of the isolates for which a dominant *var* transcript was able to be identified, DBLα tags were cloned and expressed in BL21 (DE3) pLysS *E*.*coli* cells to give recombinant proteins (‘antigens’) as previously described [50] (S1 Table).

### Sero-reactivity to recombinant proteins

Each antigen was screened for sero-reactivity to each of 36 plasma (‘antisera’) collected at each of the three timepoints (A, C1 and C2). Thirty-two of these plasma samples came from the same children from which the 36 recombinant proteins derived. Sero-reactivity was measured by coating 96-well Nunc^TM^ Maxisorb^TM^ immunoplates plates overnight at 4°C with recombinant protein diluted in Tris-buffered saline (TBS) at a concentration of 1μg/ml. Plates were blocked with 3% bovine serum albumin (BSA) in TBS, washed and then incubated in duplicate at a 1:200 dilution of individual sera diluted in 1% non-fat milk powder in TBS. After incubation for 2h at room temperature, plates were washed, then bound antibody was detected with mouse anti-human IgG alkaline phosphatase antibody or AffiniPure donkey anti-human IgM alkaline phosphatase antibody (Jackson ImmunoResearch Laboratories, Inc.) diluted 1:5000 in 1% non-fat milk powder in TBS. The reaction was developed using *o*-Phenylenediamine dihydrochloride (Sigma-Aldrich) and then stopped with 2M sulphuric acid. Optical density (OD) was read at 450nm in an ELISA microplate reader (BioTek Synergy 4, BioTek Instruments).

### Statistical analysis

Prior to building antigen and serological maps, sero-reactivity levels (log_10_ OD values) were explored for systematic effects of timepoint, DBLα-tag antigen and antisera by conducting analysis of variance fitting a series of mixed linear models in the *lme4* package in R [52]. Model 1 included a fixed effect for timepoint (sometimes fitted as a fixed-level factor and sometimes as a continuous variable) and random effects for antigen and antisera within antigen: the latter term thus tested for interactions between antigens and antisera. Model 2 was as for Model 1 but substituting antisera within antigen with a fixed effect for ‘heterology’, i.e., whether the antigen-antisera pair was from the same patient (homologous) or not (heterologous). Model 3, which was only applied to data from heterologous antigen-antisera pairs, fitted fixed effects for timepoint, serological cluster of the antisera (see below), and serological cluster of the antigen, with all possible interactions between these, and random effects for antigen and antisera. Model 4, which was only applied to data from homologous antigen-antisera pairs, fitted fixed effects for timepoint, serological cluster of the antisera, the interaction between these and a random effect for antisera. Model 5 fitted fixed effects for timepoint and antigen cluster (see below) and random effects for antigen and antisera. Models 6 and 7 were as for Model 5 but fitting genetic cluster (see below) or clinical group instead of antigen cluster. Means for fixed effects after adjusting for other fixed and random effects (least-squares means) were computed using the lsmeans package in R [53]. Pairwise contrasts between fixed effects of interest were formed using the same package with no adjustment for multiple testing. The variation due to antigen, antisera and their interaction was described by the ratio of the corresponding variance estimate (from the random effect) to the total variance remaining after accounting for fixed effects. Significance levels of all fixed and random effects were determined by likelihood ratios from models with and without the term of interest included. Estimates presented are from models in which interaction terms with P > 0.05 were removed.

Associations between categorical variables (disease severity class (‘clinical group’), antigen cluster, serological cluster and genetic cluster, as defined by maps (see below)) and normally distributed host and infection-related variables (parasite density, age, date of collection at the time of recruitment) were tested for significance using F-tests in a fixed effect analysis of variance with the latter as the dependent variables. Associations between categorical variables and non-normally distributed traits (DNA sequence global population frequency, homology block scores, relative gene expression levels for domain cassettes, see section below on genetic characteristics) were tested for significance using a two-sided Wilcoxon rank sum non-parametric test implemented by the ‘wilcox.test’ function in the R stats package [54]. Tests for relationships between clusters and categorical variables were performed using chi-squared tests of association using the ‘chisq.test’ function in the R stats package with Monte Carlo simulation for computation of P-values [54].

### Antigen and antisera maps

Antisera ‘maps’ (a representation of pairwise antisera ‘distances’, with respect to the antigens they ‘read’ antigens) were generated from the 36 x 36 matrix of sero-reactivity data using multidimensional scaling (MDS) implemented by the ‘cmdscale’ function in the stats package of R [54]. The elements of the distance matrix used as input for MDS were computed as (*d*_*ij*_/*d*_*max*_)^2^ where *d*_*ij*_ is the Euclidean distance between the vector of sero-reactivities of antisera pairs *i* and *j* and *d*_*max*_ is the maximum Euclidean distance for all pairs, calculated using the ‘adjacency’ function in the *WGCNA* package in R [55]. Maps were fitted in two dimensions and constructed from data with and without adjusting for mean differences in sero-reactivity across antigens of each antisera. Two further aids to visualization of antisera diversity were used. First, heatmaps of the distance matrix were created using the pheatmap package in R [56] and plotted with dendrograms based on hierarchical clustering (‘hclust’ function using the ‘average’ method in the R stats package [54]. Second, networks were constructed from the lowest 20% of distances in the distance matrix using the Davidson-Harel algorithm in the igraph package in R [57]. Antigen maps were similarly constructed but based on distances between pairs of antigens instead of antisera.

Note that the application of ‘antigenic cartography’ here differs from its original use [24,25] in that the plasma were polyclonal rather than monoclonal due to prior history of malaria in study children and the fact that multiple PfEMP1 types are expressed within the lifetime of a single infection. We did not attempt to adjust for previously existing levels of antibodies since our main interest was to relate existing antisera profiles at the time of infection, rather than responses to the current infection, to disease severity.

Genetic maps were constructed by MDS of the distance matrix based on amino acid sequence similarities after alignment using the clustalw algorithm implemented in the Geneious software [58] with gap open and extension penalties of 12 and 3, respectively. The genetic distance matrix was calculated using the ‘seqidentity’ function in the bio3d package in R [59].

Antigenic, serological and genetic clusters were defined by a combination of visual inspection of MDS maps and hierarchical clustering of the distance matrices.

### Genetic characteristics–PoLV/Cys classification, homology blocks, cassette domains, upstream promoter type and population frequencies

Antigens were categorized based on their amino acid sequence into six ‘PoLV/Cys2’ groups (Groups 1 to 6) based on motifs at four ‘positions of limited variability’ (PoLV) within the DBLα domain and by number of cysteine residues (Cys) - 2 vs. not-2—in the region between the third and fourth PoLV (PolV3 and PolV4) as described previously [10,51]. The two major groups defined by number of cysteines (Groups 1–3 vs. Groups 4–6) further divide into those containing a REY vs. non-REY motif at the second PoLV (PoLV2) (Groups 2 and 5 vs. Groups 1, 3, 4 and 6). REY types are shorter than non-REY types and are further distinct in their sequence between PoLV1 and PoLV2 [10]. Antigens were also classified for the presence of predicted ‘homology blocks’ by analyzing the DBLα-tag sequences on the “varDom” server [9,31]. The presence of specific domain cassettes, particularly (DC) 8 and 13—combinations of protein sequences from the DBLα, DBLα, DBLα and CIDR domains of PfEMP1 that have been previously strongly associated with severe malaria [14,20]–were determined using real-time quantitative PCR for each parasite isolate as described previously [14]. Upstream promoter type (A, B or C) of the antigens was likewise determined by real-time PCR [9,14]. Nucleotide sequences of each DBLα-tag was blasted against the DNA sequence in the global *var* gene database [30]. The number of *var* genes in the database which contained DBLα-tag nucleotide stretches with 95% or more identity to each DBLα-tag sequence was counted to estimate the ‘global population frequency’ of the sequence.

### Mapping of antigen and serological diversity to genetic sub-regions

To determine whether some sub-regions of the DBLα-tag sequence might better explain antigenic or antisera diversity than others, a sliding window analysis was performed in which a genetic sub-map built from sequence similarity based on windows of 14 amino acids, each offset by one, was assessed for concordance with the consensus antigenic or antisera map. Concordance was statistically evaluated by fitting a linear model to the distance matrix of the genetic sub-map with antigen or serological cluster as a fixed effect using the ‘adonis’ multivariate analysis of variance function in the vegan package in R [60]. 1200 permutations of the data were performed to determine significance levels. Concordance between genetic and antigenic or antisera maps was also assessed by computing the sum of squared differences (SS) between point locations in the two maps after rotating and scale transforming the antigenic or antisera map such that it minimized the SS, as implemented by the ‘procrustes’ function in the vegan package in R [60]. The P-value for observed goodness-of-fit statistic, R^2^ (= 1-SS), was compared to that of its empirical distribution generated from 1200 random permutations of the antigenic or antisera map using the ‘protest’ function in the vegan package This second statistical test was applied to maps built both across and within antigenic or antisera clusters.

## Supporting information

S1 Table Accession numbers for antigens. (DOCX)

S1 Fig Heatmaps of DBLα-tag antigen by antisera reactivity patterns for IgG and IgM by timepoint. Heatmap colours show strength of sero-reactivity between DBLα-tag antigens (rows) and antisera (columns) (red, high reactivity; blue, low reactivity) at the acute (**a, b**), C1 (**c, d**) and C2 (**e, f**) time points for IgG (**a**, **c**, **e**) and IgM (**b**, **d**, **f**). Colours in side bars indicate clinical group, serological cluster, antigen cluster, genetic cluster, PolV group (legends in centre) and individual antigen/antisera. Row and column orders for all panels are the same as in (**a**) and in Fig 1 of the main text. Asterisks mark ‘indicator antigens’ (see main text). Data are not pre-adjusted for mean antisera and mean antigen levels.
(TIF)

S2 Fig Mean reactivity of DBLα-tag antigens by timepoint. Mean reactivity (measured in log_10_ OD units) across 36 antisera for each of 36 DBLα-tag antigens (circular symbols, one colour per antigen, as in S1 Fig) at the time of acute disease (x-axis) vs. that at the first (C1) and second convalescent (C2) timepoints (y-axes) for IgG (panels **a** and **b**) and IgM (panels **c** and **d**) (y-axes). The dashed horizontal line shows the 95% confidence limit for reactivity of the 36 antigens to 8 sera from Europeans with no history of infection with *P*. *falciparum*.
(TIF)

S3 Fig Mean reactivity of antisera by timepoint. Mean reactivity (measured in log_10_ OD units) across 36 DBLα-tag antigens for each of 36 antisera (circular symbols, one colour per serum) at the time of acute disease (x-axis) vs. that at the first (C1) and second convalescent (C2) timepoints (y-axes) for IgG (panels **a** and **b**) and IgM (panels **c** and **d**) (y-axes). Antisera showing abnormally low responses at a single timepoint are labelled in black text. The dashed horizontal line shows the 95% confidence limit for reactivity of the 36 antigens to 8 sera from Europeans with no history of infection with *P*. *falciparum*.
(TIF)

S4 Fig Heatmaps and network representations of antisera similarity based on IgG responses. Heatmap colours show degree of similarity between antisera (red, most similar; blue, most distant) based on IgG responses at the acute (**a**), C1 (**c**) and C2 (**e**) stages. Colours in side bars indicate clinical group, serological cluster, antigen cluster, genetic cluster, PoLV group (legends in centre) and individual antisera. Asterisks mark antisera derived from the same host as indicator antigens. Network plots for IgG responses at the acute (**b**), C1 (**d**) and C2 (**f**) stages were based on the adjacency matrix after adjusting for mean differences between antisera (see Methods).
(TIF)

S5 Fig Heatmaps and network representations of antisera similarity based on IgM responses. Heatmap colours show degree of similarity between antisera (red, most similar; blue, most distant) based on IgM responses at the acute (**a**), C1 (**c**) and C2 (**e**) stages. Colours in side bars indicate clinical group, serological cluster, antigen cluster, genetic cluster, PoLV group (legends in centre) and individual antisera. Asterisks mark antisera derived from the same host as indicator antigens. Network plots for IgM responses at the acute (**b**), C1 (**d**) and C2 (**f**) stages were based on the adjacency matrix after adjusting for mean differences between antisera (see Methods).
(TIF)

S6 Fig DBLα-tag antigen maps for IgG and IgM responses. Maps were constructed based on IgG (**a**) and IgM (**b**) sero-reactivity data at the acute timepoint. Point size is proportional to average sero-reactivity for the DBLα-tag antigen across all three time points. Point colours indicate antigen cluster. Colours of outer circles indicate serological cluster. Asterisks mark indicator antigens.
(TIF)

S7 Fig Heatmaps and network representations of DBLα-tag antigen similarity based on IgG responses. Heatmap colours show degree of similarity between DBLα-tag antigens (red, most similar; blue, most distant) based on IgG responses at the acute (**a**), C1 (**c**) and C2 (**e**) stages. Colours in side bars indicate clinical group, serological cluster, antigen cluster, genetic cluster, PolV group (legends in centre) and individual antigens. Asterisks mark indicator antigens. Network plots for IgG responses at the acute (**b**), C1 (**d**) and C2 (**f**) stages were based on the adjacency matrix after adjusting for mean differences between antigens (see Methods).
(TIF)

S8 Fig Heatmaps and network representations of DBLα-tag antigen similarity based on IgM responses. Heatmap colours show degree of similarity between DBLα-tag antigens (red, most similar; blue, most distant) based on IgM responses at the acute (**a**), C1 (**c**) and C2 (**e**) stages. Colours in side bars indicate clinical group, serological cluster, antigen cluster, genetic cluster, PolV group (legends in centre) and individual antigens. Asterisks mark indicator antigens. Network plots for IgM responses at the acute (**b**), C1 (**d**) and C2 (**f**) stages were based on the adjacency matrix after adjusting for mean differences between antigens (see Methods).
(TIF)

S9 Fig Genetic window sub-maps with strongest correspondence to serological, antigen and genetic clusters. Genetic maps based on genetic similarity among isolates in DBLα regions for windows with midpoints at **a**, position 47; **b**, position 69; **c**, position 45; **d**, position 112. Sub-maps in **a** and **b** are those which correspond most (highest R^2^, values given in black text with P-values) with serological clusters; sub-maps in **c** and **d** correspond best with antigen and genetic clusters (Fig 6B), respectively. Colours of open symbols indicate serological, antigen and genetic clusters (legends on right). In **b**, closed symbols indicate PoLV grouping. **e**, Amino acid sequences in the region of the DBLα domain showing strongest correspondence with serological and antigen clusters. Horizontal dashed lines indicate the windows used for constructing the maps in **a**, **b** and **c** above. Membership of clusters and PoLV groups are indicated in black text to the left of the figure. Black closed circles mark the antigens with the REY motif at the PoLV2 position (grey bar, Groups 2 and 5) which are highly concentrated in isolates causing severe disease (Serological Cluster II). Asterisks mark indicator antigens. Coloured bars at the top of the figure indicate locations of homology blocks (see Fig 7 in main text). The horizontal black line indicates the DBLα sub-domain defined as S2b by Rask et al. (2010) with the arrowhead indicating that it extends beyond the window shown.
(TIF)
