# Correction: Antigenic cartography of immune responses to *Plasmodium falciparum* erythrocyte membrane protein 1 (PfEMP1)

**DOI:** 10.1371/journal.ppat.1008018

**Published:** 2019-08-15

**Authors:** The PLOS Pathogens Staff

There are a number of errors in the caption for Fig 2, “Antisera maps for IgG and IgM" that arose during typesetting. Please see the complete, correct Fig 2 caption here. The publisher apologizes for the errors.

**Fig 2:**
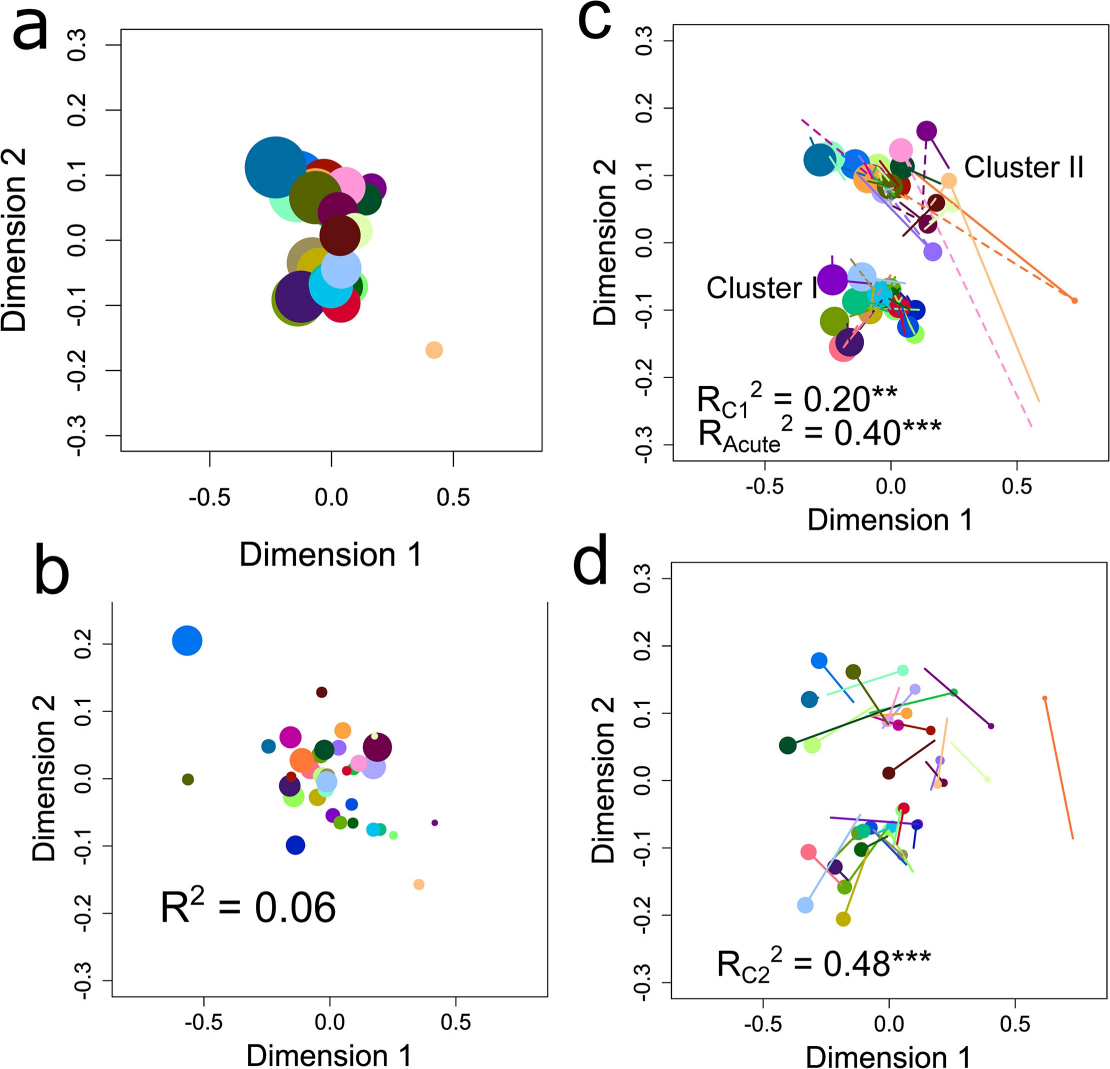
Antisera maps for IgG and IgM. Maps were constructed from data on **a**, IgG at the acute timepoint; **b**, IgM at the acute timepoint; **c**, IgG at timepoint C2 (the consensus antisera map); **d**, IgM at timepoint C2. In **c**, solid and dashed lines point to positions in the map for IgG at the acute (as in **a**) and C1 timepoints, respectively. In **d**, solid lines point to positions in the consensus antisera map shown by the points in **c**. Symbol size is proportional to the antisera’s average log_10_ OD value at the timepoint indicated. R^2^ values represent goodness-of-fit to the consensus map with subscripts indicating the timepoint of the map being compared and asterisks indicating significance (***, P < 0.001; **, P < 0.01).
