# Supplementary material for: Antigenic cartography of immune responses to Plasmodium falciparum erythrocyte membrane protein 1 (PfEMP1)
Source: PLoS Pathog. 2019 Jul 1;15(7):e1007870. doi: 10.1371/journal.ppat.1007870 (PMC6625739; doi:10.1371/journal.ppat.1007870)
Supplement: S1 Table — (DOCX) [file ppat.1007870.s001.docx]

# Table S1. Accession numbers for antigens.

| **Isolate name in this study** | **Isolate name in EMBL** | **EMBL accession no.** |
| --- | --- | --- |
| 6387 | 6387 | FR874861.1 |
| 6408 | 6408 | FR874863.1 |
| 6429 | 6429 | FR874864.1 |
| 6430 | 6430 | FR874865.1 |
| 6433 | 6433 | FR874866.1 |
| 6485 | 6485 | FR874867.1 |
| 6964 | 6964 | FR874868.1 |
| 7045 | 7045 | FR874869.1 |
| 7069 | 7069 | FR874870.1 |
| 7116 | 7116 | FR874872.1 |
| 7134 | 7134 | FR874873.1 |
| 7157 | 7157 | FR874874.1 |
| 7160 | 7160 | FR874875.1 |
| 7183 | 7183 | FR874876.1 |
| 7198 | 7198 | FR874877.1 |
| 7204 | 7204 | FR874878.1 |
| 7249 | 7249 | FR874879.1 |
| 7250 | 7250.1 | LR594710 |
| 7323 | 7323 | FR874881.1 |
| 7337 | 7337 | FR874882.1 |
| 7391 | 7391 | FR874883.1 |
| 7410 | 7410.1 | LR594711 |
| 7506 | 7250 | FR874880.1 |
| 7530 | 7530 | FR874886.1 |
| 7630 | 7630 | FR874887.1 |
| 7781 | 7781 | FR874888.1 |
| 7799 | 7799 | FR874889.1 |
| 7860 | 7860 | FR874890.1 |
| 7864 | 7864 | FR874891.1 |
| 8204 | 8204 | FR874892.1 |
| 8344 | 8344 | FR874893.1 |
| 8349 | 8349 | FR874894.1 |
| 8383 | 8383 | FR874895.1 |
| 8477 | 8477 | FR874897.1 |
| 8482 | 8482 | FR874898.1 |
| 8585 | 8585 | FR874899.1 |
| 8706 | 8618 | FR874900.1 |
