# Supplementary figures and images for: Antigenic cartography of immune responses to Plasmodium falciparum erythrocyte membrane protein 1 (PfEMP1)
Source: PLoS Pathog. 2019 Jul 1;15(7):e1007870. doi: 10.1371/journal.ppat.1007870 (PMC6625739; doi:10.1371/journal.ppat.1007870)

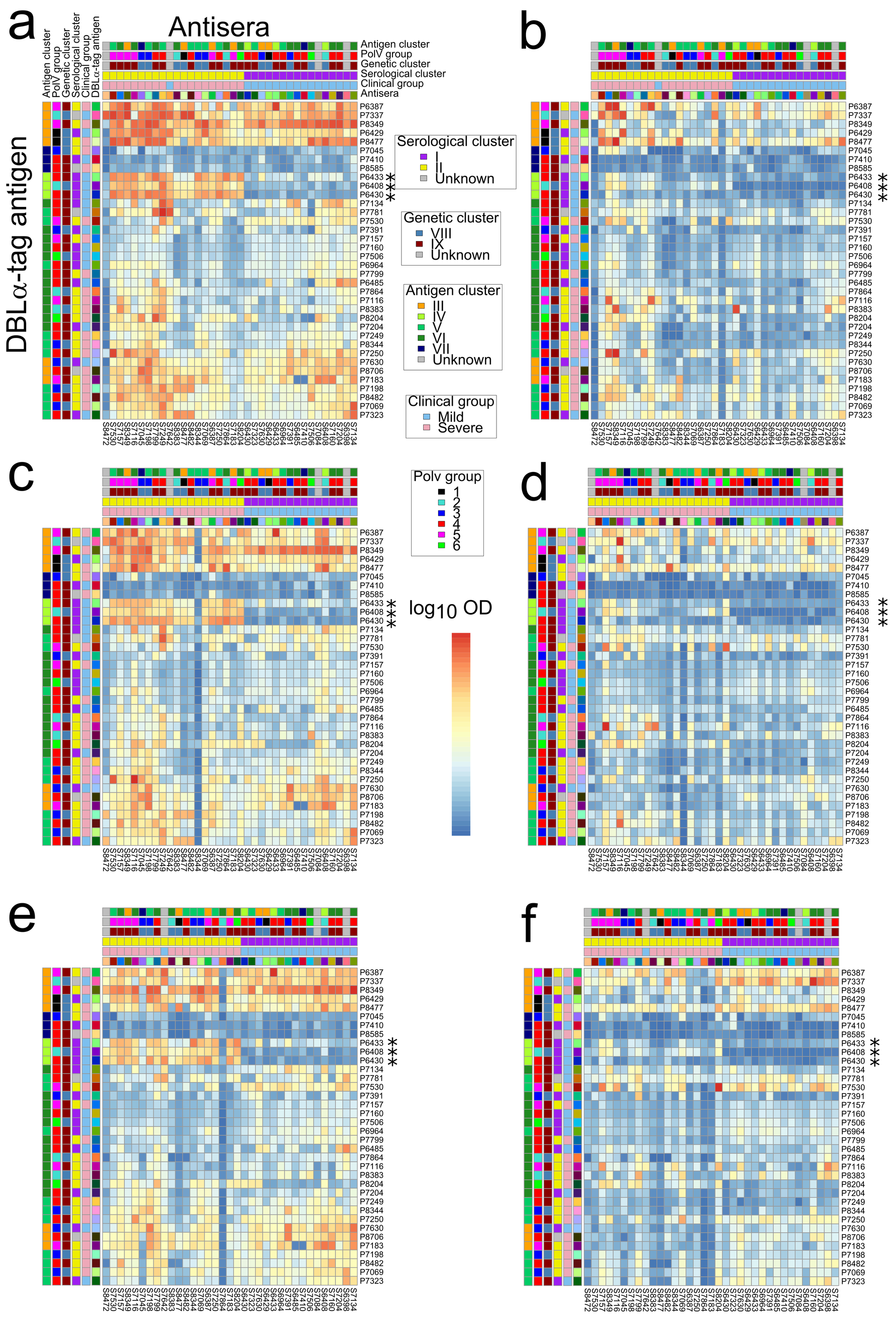

Supplement: S1 Fig — Heatmap colours show strength of sero-reactivity between DBLα-tag antigens (rows) and antisera (columns) (red, high reactivity; blue, low reactivity) at the acute (a, b), C1 (c, d) and C2 (e, f) time points for IgG (a, c, e) and IgM (b, d, f). Colours in side bars indicate clinical group, serological cluster, antigen cluster, genetic cluster, PolV group (legends in centre) and individual antigen/antisera. Row and column orders for all panels are the same as in (a) and in Fig 1 of the main text. Asterisks mark ‘indicator antigens’ (see main text). Data are not pre-adjusted for mean antisera and mean antigen levels. (TIF) [file ppat.1007870.s002.tif]

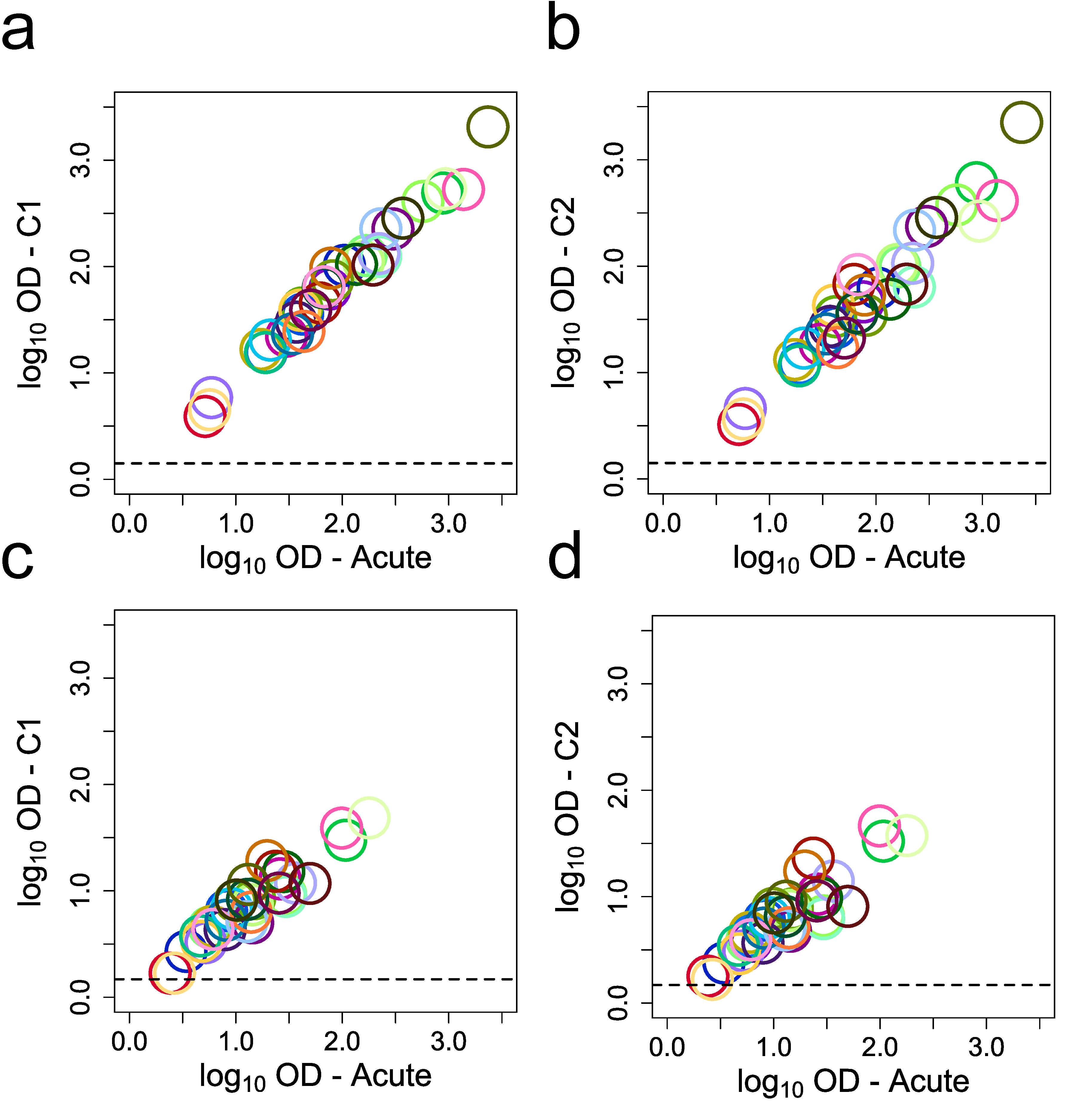

Supplement: S2 Fig — Mean reactivity (measured in log10 OD units) across 36 antisera for each of 36 DBLα-tag antigens (circular symbols, one colour per antigen, as in S1 Fig) at the time of acute disease (x-axis) vs. that at the first (C1) and second convalescent (C2) timepoints (y-axes) for IgG (panels a and b) and IgM (panels c and d) (y-axes). The dashed horizontal line shows the 95% confidence limit for reactivity of the 36 antigens to 8 sera from Europeans with no history of infection with P. falciparum. (TIF) [file ppat.1007870.s003.tif]

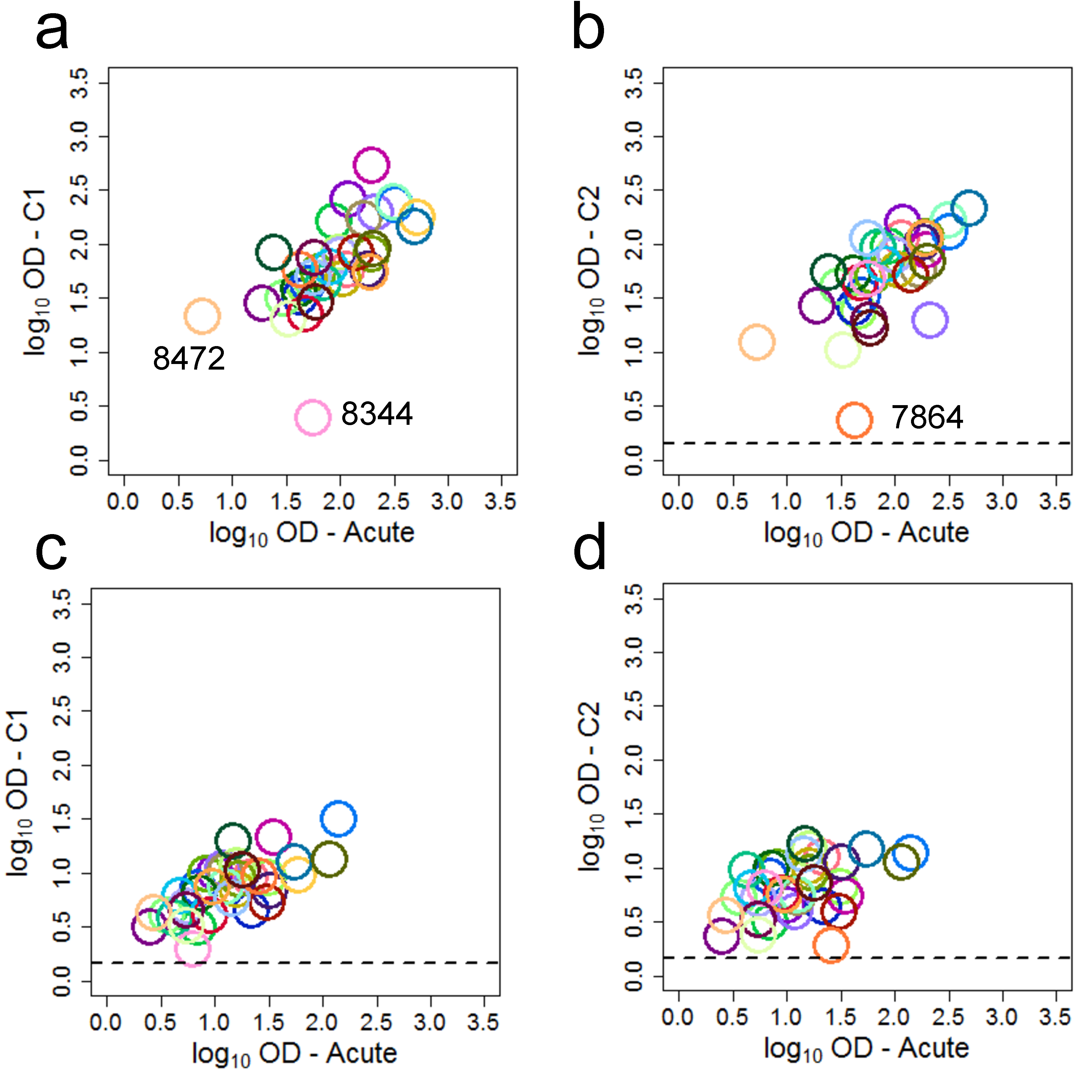

Supplement: S3 Fig — Mean reactivity (measured in log10 OD units) across 36 DBLα-tag antigens for each of 36 antisera (circular symbols, one colour per serum) at the time of acute disease (x-axis) vs. that at the first (C1) and second convalescent (C2) timepoints (y-axes) for IgG (panels a and b) and IgM (panels c and d) (y-axes). Antisera showing abnormally low responses at a single timepoint are labelled in black text. The dashed horizontal line shows the 95% confidence limit for reactivity of the 36 antigens to 8 sera from Europeans with no history of infection with P. falciparum. (TIF) [file ppat.1007870.s004.tif]

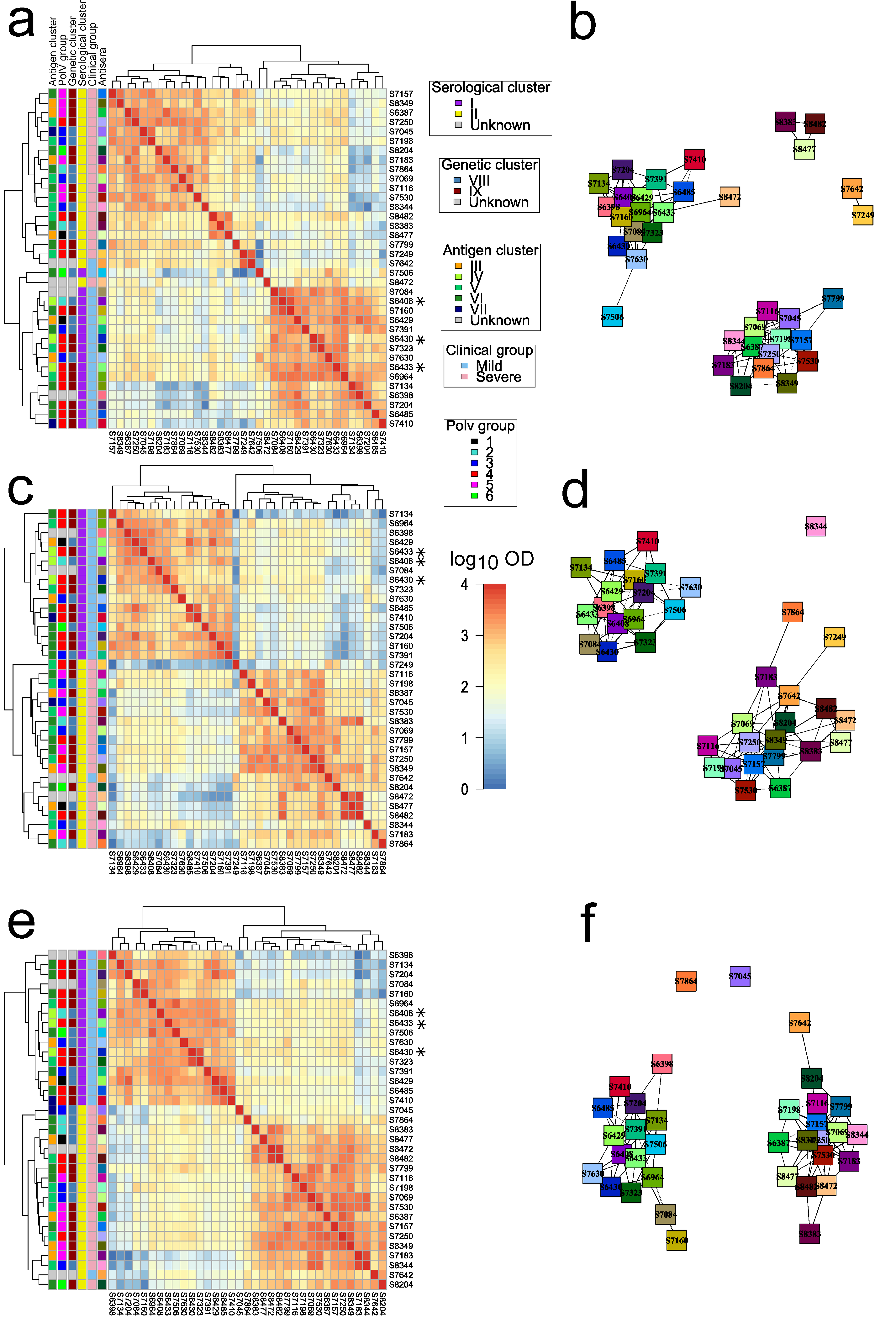

Supplement: S4 Fig — Heatmap colours show degree of similarity between antisera (red, most similar; blue, most distant) based on IgG responses at the acute (a), C1 (c) and C2 (e) stages. Colours in side bars indicate clinical group, serological cluster, antigen cluster, genetic cluster, PoLV group (legends in centre) and individual antisera. Asterisks mark antisera derived from the same host as indicator antigens. Network plots for IgG responses at the acute (b), C1 (d) and C2 (f) stages were based on the adjacency matrix after adjusting for mean differences between antisera (see Methods). (TIF) [file ppat.1007870.s005.tif]

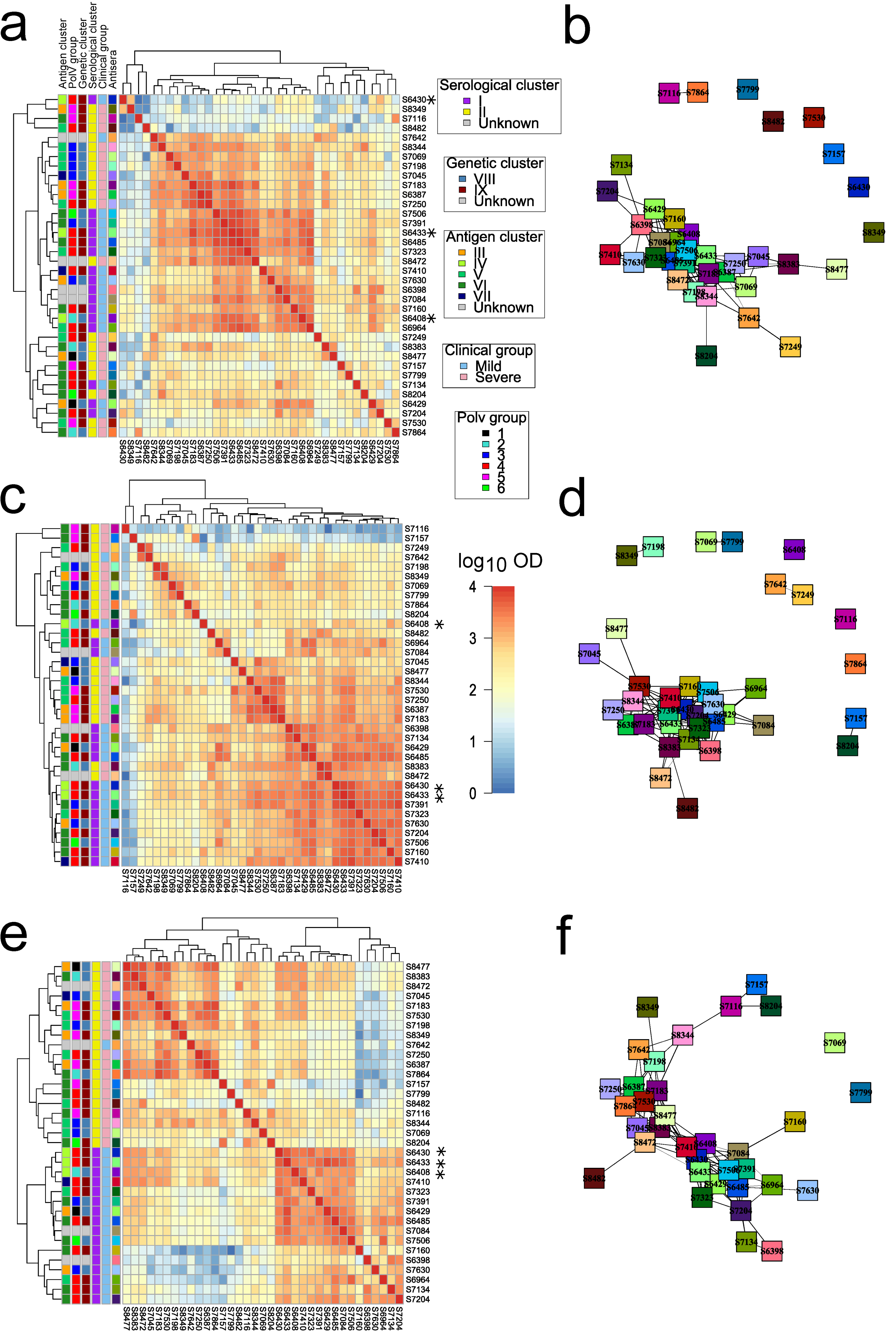

Supplement: S5 Fig — Heatmap colours show degree of similarity between antisera (red, most similar; blue, most distant) based on IgM responses at the acute (a), C1 (c) and C2 (e) stages. Colours in side bars indicate clinical group, serological cluster, antigen cluster, genetic cluster, PoLV group (legends in centre) and individual antisera. Asterisks mark antisera derived from the same host as indicator antigens. Network plots for IgM responses at the acute (b), C1 (d) and C2 (f) stages were based on the adjacency matrix after adjusting for mean differences between antisera (see Methods). (TIF) [file ppat.1007870.s006.tif]

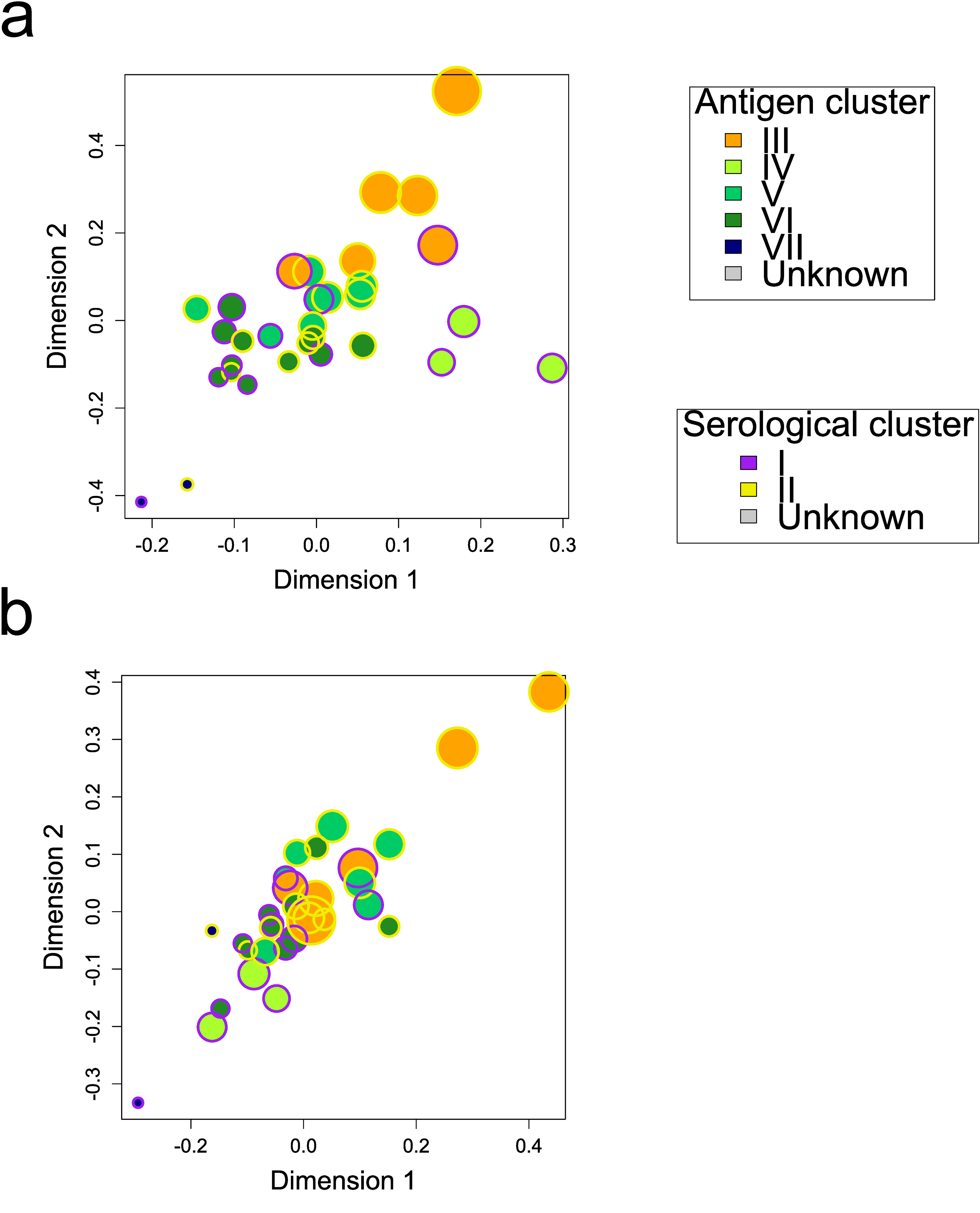

Supplement: S6 Fig — Maps were constructed based on IgG (a) and IgM (b) sero-reactivity data at the acute timepoint. Point size is proportional to average sero-reactivity for the DBLα-tag antigen across all three time points. Point colours indicate antigen cluster. Colours of outer circles indicate serological cluster. Asterisks mark indicator antigens. (TIF) [file ppat.1007870.s007.tif]

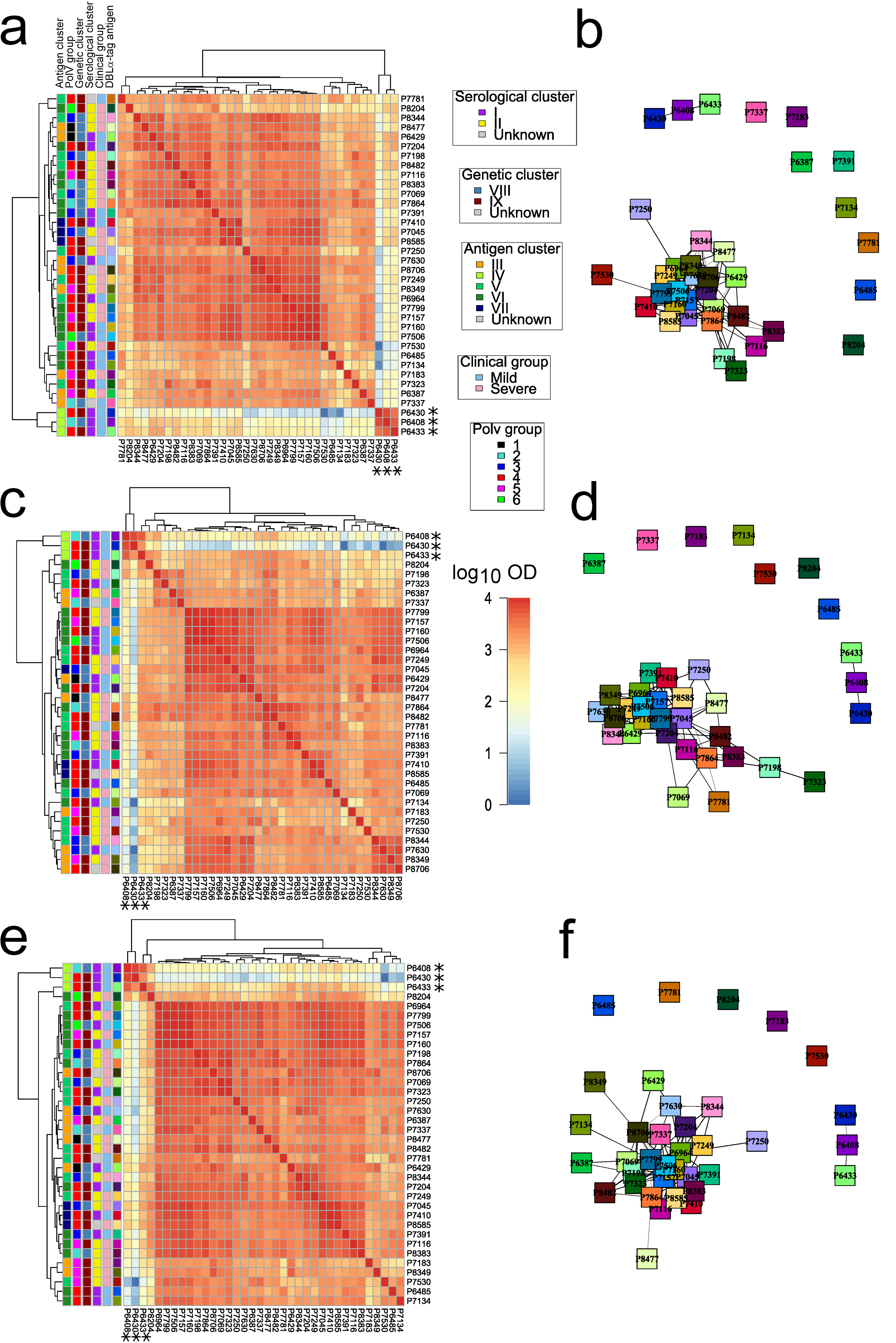

Supplement: S7 Fig — Heatmap colours show degree of similarity between DBLα-tag antigens (red, most similar; blue, most distant) based on IgG responses at the acute (a), C1 (c) and C2 (e) stages. Colours in side bars indicate clinical group, serological cluster, antigen cluster, genetic cluster, PolV group (legends in centre) and individual antigens. Asterisks mark indicator antigens. Network plots for IgG responses at the acute (b), C1 (d) and C2 (f) stages were based on the adjacency matrix after adjusting for mean differences between antigens (see Methods). (TIF) [file ppat.1007870.s008.tif]

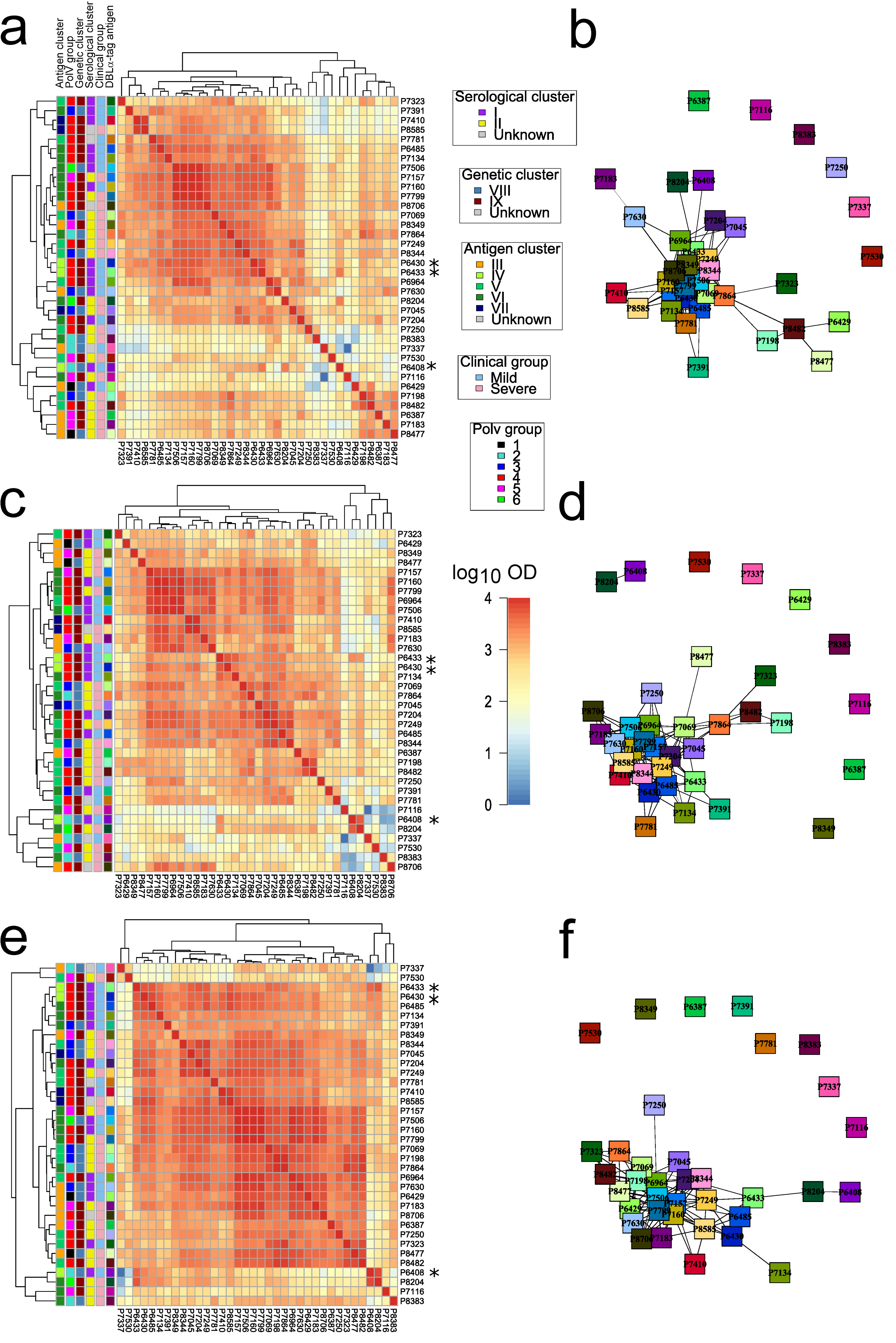

Supplement: S8 Fig — Heatmap colours show degree of similarity between DBLα-tag antigens (red, most similar; blue, most distant) based on IgM responses at the acute (a), C1 (c) and C2 (e) stages. Colours in side bars indicate clinical group, serological cluster, antigen cluster, genetic cluster, PolV group (legends in centre) and individual antigens. Asterisks mark indicator antigens. Network plots for IgM responses at the acute (b), C1 (d) and C2 (f) stages were based on the adjacency matrix after adjusting for mean differences between antigens (see Methods). (TIF) [file ppat.1007870.s009.tif]

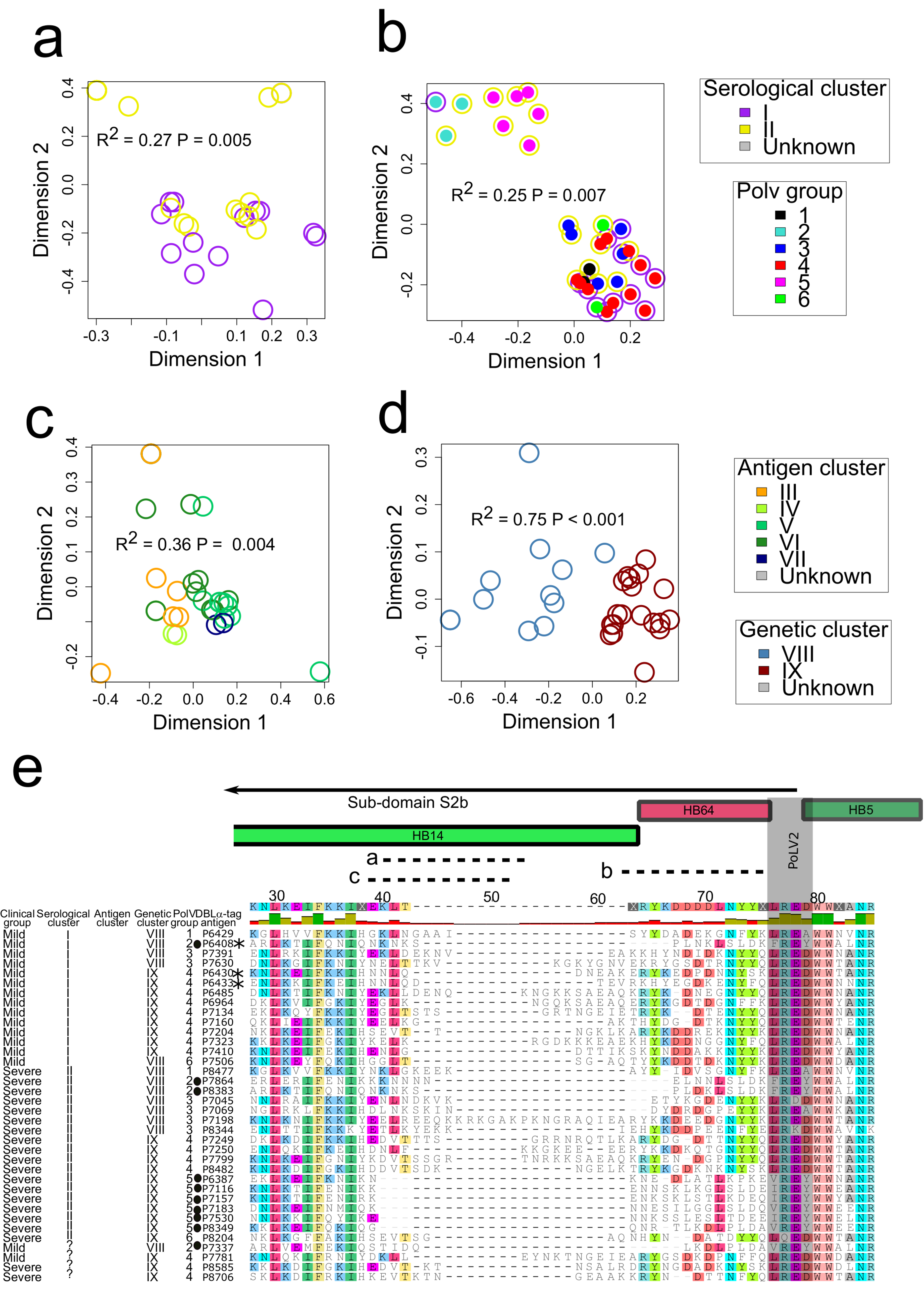

Supplement: S9 Fig — Genetic maps based on genetic similarity among isolates in DBLα regions for windows with midpoints at a, position 47; b, position 69; c, position 45; d, position 112. Sub-maps in a and b are those which correspond most (highest R2, values given in black text with P-values) with serological clusters; sub-maps in c and d correspond best with antigen and genetic clusters (Fig 6B), respectively. Colours of open symbols indicate serological, antigen and genetic clusters (legends on right). In b, closed symbols indicate PoLV grouping. e, Amino acid sequences in the region of the DBLα domain showing strongest correspondence with serological and antigen clusters. Horizontal dashed lines indicate the windows used for constructing the maps in a, b and c above. Membership of clusters and PoLV groups are indicated in black text to the left of the figure. Black closed circles mark the antigens with the REY motif at the PoLV2 position (grey bar, Groups 2 and 5) which are highly concentrated in isolates causing severe disease (Serological Cluster II). Asterisks mark indicator antigens. Coloured bars at the top of the figure indicate locations of homology blocks (see Fig 7 in main text). The horizontal black line indicates the DBLα sub-domain defined as S2b by Rask et al. (2010) with the arrowhead indicating that it extends beyond the window shown. (TIF) [file ppat.1007870.s010.tif]
